# Convergent consequences of parthenogenesis on stick insect genomes

**DOI:** 10.1126/sciadv.abg3842

**Published:** 2022-02-23

**Authors:** Kamil S. Jaron, Darren J. Parker, Yoann Anselmetti, Patrick Tran Van, Jens Bast, Zoé Dumas, Emeric Figuet, Clémentine M. François, Keith Hayward, Victor Rossier, Paul Simion, Marc Robinson-Rechavi, Nicolas Galtier, Tanja Schwander

**Affiliations:** 1 Department of Ecology and Evolution, University of Lausanne, Lausanne, Switzerland.; 2 Swiss Institute of Bioinformatics, Lausanne, Switzerland.; 3 Institute of Evolutionary Biology, School of Biological Sciences, University of Edinburgh, Edinburgh EH9 3FL, UK.; 4 ISEM—Institut des Sciences de l’Evolution, Montpellier, France.

## Abstract

The shift from sexual reproduction to parthenogenesis has occurred repeatedly in animals, but how the loss of sex affects genome evolution remains poorly understood. We generated reference genomes for five independently evolved parthenogenetic species in the stick insect genus *Timema* and their closest sexual relatives. Using these references and population genomic data, we show that parthenogenesis results in an extreme reduction of heterozygosity and often leads to genetically uniform populations. We also find evidence for less effective positive selection in parthenogenetic species, suggesting that sex is ubiquitous in natural populations because it facilitates fast rates of adaptation. Parthenogenetic species did not show increased transposable element (TE) accumulation, likely because there is little TE activity in the genus. By using replicated sexual-parthenogenetic comparisons, our study reveals how the absence of sex affects genome evolution in natural populations, providing empirical support for the negative consequences of parthenogenesis as predicted by theory.

## Teaser

The convergent evolution of parthenogenesis in stick insects results in heterozygosity loss and slower adaptive evolution.

## INTRODUCTION

Sex: What is it good for? The reason why most eukaryotes take a complicated detour to reproduction, when more straightforward options, such as parthenogenesis, are available, remains a central and largely unanswered question in evolutionary biology (*1*, *2*). Animal species in which parthenogenetic reproduction is the sole form of replication typically occur at the tips of phylogenies, and only a few of them have succeeded as well as their sexually reproducing relatives (*3*). In other words, most parthenogenetic lineages may eventually be destined for extinction. These incipient evolutionary failures, however, are invaluable as by understanding their fate something may be learned about the adaptive value of sex.

Parthenogenesis is thought to be favored in the short term because it generates a transmission advantage (*4*, *5*), as well as the advantage of assured reproduction when mates are scarce (*6*, *7*). The short-term benefits of parthenogenesis, however, are believed to come along with long-term costs. For example, the physical linkage between loci it entails can generate interferences that decrease the efficacy of natural selection [e.g., (*8*–*10*), reviewed in (*11*)]. This is expected to translate into reduced rates of adaptation and increased accumulation of mildly deleterious mutations, which may potentially drive the extinction of parthenogenetic lineages.

In addition to these predicted effects on adaptation and mutation accumulation, parthenogenesis is expected to drive major aspects of genome evolution. A classical prediction is that heterozygosity (i.e., intraindividual polymorphism) increases over time in the absence of recombination, as the two haploid genomes diverge independently of each other, generating the so-called “Meselson effect” (*12*, *13*). Parthenogenesis can also affect the dynamics of transposable elements (TEs), resulting in either increased or decreased genomic TE loads (*14*, *15*). Last, some forms of parthenogenesis might facilitate the generation and maintenance of structural variants (SVs), which in sexuals are counter-selected due to the constraints of properly pairing homologous chromosomes during meiosis (*16*).

**Fig. 1.**
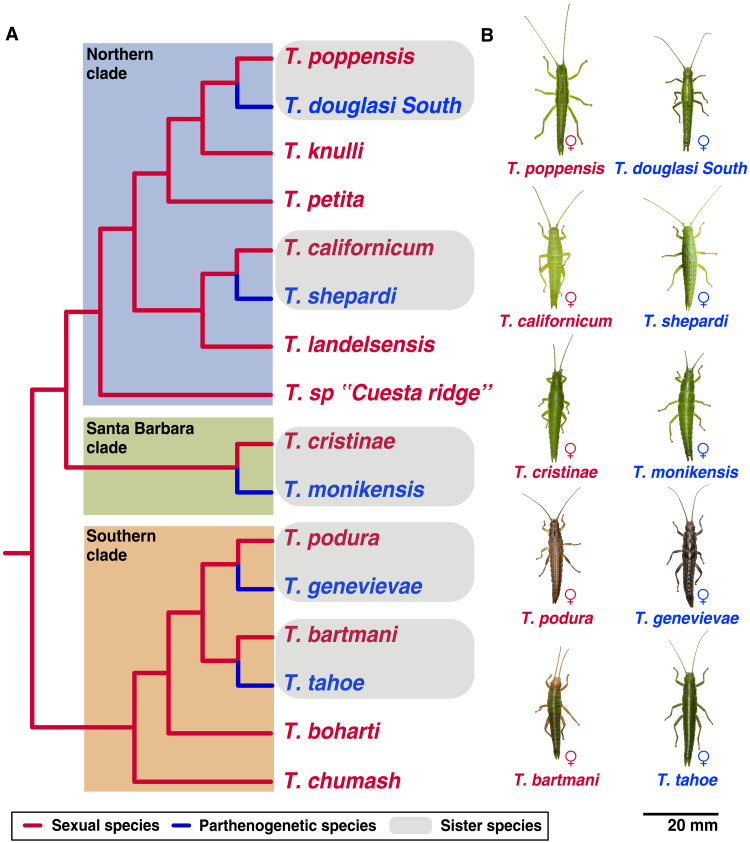
Multiple, independent transitions from sexual to parthenogenetic reproduction are known in the genus *Timema* , each representing a biological replicate of parthenogenesis, and with a close sexual relative at hand for comparison. (*31*) (**A**) Phylogenetic relationships of *Timema* species [adapted from (*31*, *45*)]. (**B**) Species sequenced in this study. Photos taken by Bart Zijlstra (www.bartzijlstra.com).

We tested these predictions by comparing the genomes of five independently derived parthenogenetic stick insect species in the genus *Timema* with their close sexual relatives (Fig. 1). These replicate comparisons allowed us to solve the key problem in understanding the consequences of parthenogenesis for genome evolution: separating the consequences of parthenogenesis from lineage-specific effects (*16*). *Timema* are wingless, plant-feeding insects endemic to western North America. Parthenogenetic species in this genus are diploid and of nonhybrid origin (*17*) and ecologically similar to their sexual relatives. Previous research, based on a small number of microsatellite markers, has suggested that oogenesis in parthenogenetic *Timema* is functionally mitotic, as no loss of heterozygosity between females and their offspring was detected (*17*).

## RESULTS AND DISCUSSION

### De novo genome assemblies reveal extremely low heterozygosity in parthenogenetic stick insects

We generated 10 de novo genomes of *Timema* stick insects, from 5 parthenogenetic and 5 sexual species (Fig. 1 and tables S1 and S2). Genomes were subjected to quality control, screened for contamination, and annotated (see Methods and Supplementary Text). The final reference genomes were largely haploid, spanned 75 to 95% of the estimated genome size [1.38 gigabase pairs (Gbp); (*18*)], and were sufficiently complete for downstream analyses, as shown by the count of single copy orthologs conserved across insects [96% of BUSCO genes (*19*) detected on average; table S3]. A phylogeny based on a conservative set of 3975 1:1 orthologous genes (data S1) corroborated published phylogenies and molecular divergence estimates in the *Timema* genus (fig. S1). Last, we identified 55 candidate horizontal gene transfers (HGTs) from nonmetazoans. Using long reads, we were able to corroborate six of the eight HGT candidates present in one species (*T. douglasi*; see Supplementary Text), indicating that most of our HGT candidates are likely true HGTs rather than false positives. The two remaining HGT candidates in *T. douglasi* were most likely misassembled chimeric contigs. All 55 candidate HGTs occurred early on in *Timema* evolutionary history, well before the evolution of parthenogenesis in the genus (see Supplementary Text).

**Fig. 2.**
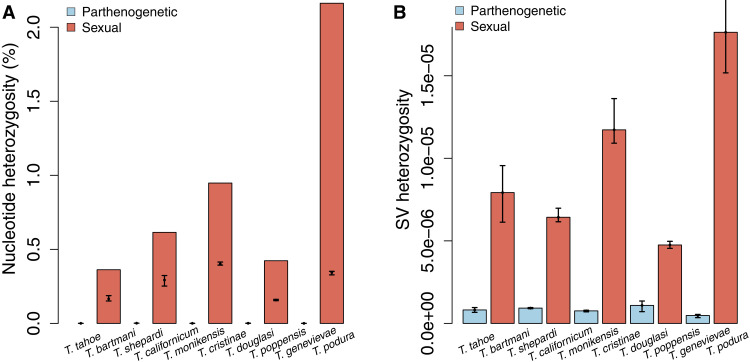
Extremely low heterozygosity in parthenogenetic *Timema* species for different types of variants. (**A**) Nucleotide heterozygosity represented by bars indicates genome-wide estimates for the reference individuals (based on raw reads, see Methods); heterozygosity based on SNP calls in resequenced individuals is indicated by points and represents a conservative estimation of heterozygosity in the assembled genome portions (with error bars indicating the range of estimates across individuals). (**B**) Heterozygous SVs (reported as number of heterozygous SVs/number of callable sites) in resequenced individuals (with error bars indicating the range of estimates across individuals). Note that although heterozygous SNPs and SVs were called using stringent parameters, it is likely that a large portion are false positives in parthenogenetic *Timema* (see Supplementary Text).

We estimated genome-wide nucleotide heterozygosity in each reference genome directly from sequencing reads, using a reference-free technique [genome profiling analysis (*20*)]. These analyses revealed extreme heterozygosity differences between the sexual and parthenogenetic species. The five sexual *Timema* featured nucleotide heterozygosities within the range previously observed in other sexual species [Fig. 2; (*21*)]. The heterozygosities in the parthenogenetic species were substantially lower, and so low that reference-free analyses could not distinguish heterozygosity from sequencing error (see Supplementary Text). We therefore compared heterozygosity between sexuals and parthenogens by calling single-nucleotide polymorphisms (SNPs) in five resequenced individuals per species. This analysis corroborated the finding that parthenogens have extremely low (<10^−5^) heterozygosity, being at least 140 times lower than that found in their sexual sister species [permutation analysis of variance (ANOVA), reproductive mode effect *P* = 0.0049; Fig. 2]. Screening for SVs (indels, tandem duplications, and inversions) in sexual and parthenogenetic individuals revealed the same pattern: extensive and variable heterozygosity in sexual species and homozygosity in the parthenogens (see Fig. 2 and Supplementary Text). Some heterozygosity in *Timema* parthenogens could be present in genomic regions not represented in our assemblies, such as centromeric and telomeric regions. These regions, however, represent a relatively small fraction of the total genome, meaning that, for most of the genome at least, *Timema* parthenogens are either largely or completely homozygous for all types of variants (see Supplementary Text).

We are able to demonstrate that extremely low heterozygosity is a convergent consequence of parthenogenesis in *Timema*. However, while extremely low heterozygosity is the rule for *Timema* parthenogens, this is not the case for all parthenogenetic species. For example, we have previously shown that some parthenogenetic animal species, particularly those of hybrid origin, are characterized by relatively high heterozygosity levels (*16*). This indicates that the consequences of parthenogenesis for heterozygosity are likely to be lineage-specific.

The unexpected finding of extremely low heterozygosity in *Timema* parthenogens raises the question of when and how heterozygosity was lost. For example, the bulk of heterozygosity could have been lost during the transition from sexual reproduction to obligate parthenogenesis (*22*). This would be the case if functionally mitotic parthenogenesis was derived from automictic parthenogenesis, meaning that recombination and meiosis take place, and diploidy is restored secondarily, for example, via fusion of two of the four meiotic products. Similar to inbreeding, automictic parthenogenesis thus results in the rapid loss of heterozygosity over time (*23*). Automixis can then be co-opted into functionally mitotic parthenogenesis via recombination suppression, and by solely fusing meiotic products separated during meiosis I and not meiosis II (*17*). Alternatively, heterozygosity loss in *Timema* parthenogens could be a continuous and ongoing process. To distinguish these options, we investigated the origin of the genetic variation present among different homozygous genotypes in each parthenogenetic species. We found that only 6 to 19% of the SNPs called in a parthenogen are at positions that are also polymorphic in the sexual relative (table S4). This means that most of the variation in parthenogens likely results from mutations that appeared after the split from the sexual lineage. Although it is possible that some of the parthenogen-specific polymorphisms may represent ancestral polymorphisms that were purged in the sexual species, it is unlikely that this would be the case for most SNPs in parthenogens. This implies that heterozygosity generated through new mutations is most likely lost continuously in parthenogens and was not solely lost at the inception of parthenogenesis. The most likely explanation for these findings is that parthenogenetic *Timema* are not functionally mitotic but automictic. Formally distinguishing between automixis and functionally mitotic parthenogenesis with gene conversion will require cell biological data, which is currently not available for *Timema*. It is important to note that the key theoretical predictions regarding the consequences of sex do not change between automixis or functionally mitotic parthenogenesis. Thus, although automixis can allow for the purging of heterozygous deleterious mutations (*24*), the classical predictions for the long-term costs of asexuality extend to automictic parthenogens because, as for obligate selfers, linkage among genes is still much stronger than in classical sexual species (*25*). This is especially the case in largely homozygous parthenogens, where recombination and segregation, even if mechanistically present, have no effect on genotype diversities.

Functional mitosis in *Timema* parthenogens was previously inferred from the inheritance of heterozygous microsatellite genotypes between females and their offspring (*17*), a technique widely used in nonmodel organisms with no cytological data available [e.g., (*26*, *27*)]. The most likely reconciliation of these results with our finding of extreme homozygosity is that heterozygosity is maintained in only a small portion of the genome, for example, the centromeres or telomeres, or between paralogs. Consistent with this idea, we were unable to locate several of the microsatellite-containing regions in even the best *Timema* genome assemblies (see Supplementary Text), suggesting that these regions are not present in our assemblies due to the inherent difficulty of assembling repetitive genome regions from short read data (*28*).

### Extensive variation in genotype diversity between parthenogenetic populations

Parthenogenesis and sexual reproduction are expected to drive notably different distributions of polymorphisms in genomes and populations. Within genomes, different regions experience different types of selection with sometimes opposite effects on the levels of polymorphisms within populations, such as purifying versus balancing selection (*29*). The increased linkage among genes in parthenogenetic as compared to sexual species is expected to homogenize diversity levels across different genome regions. At the species level, parthenogens are expected to have reduced genetic diversities relative to sexual species because of likely bottlenecks occurring during the origin of parthenogenesis. At the population level, recurrent sweeps of specific genotypes in parthenogenetic populations can lead to extremely low genetic diversity and even to the fixation of a single genotype, while sweeps in sexual populations typically reduce diversity only in specific genome regions.

**Fig. 3.**
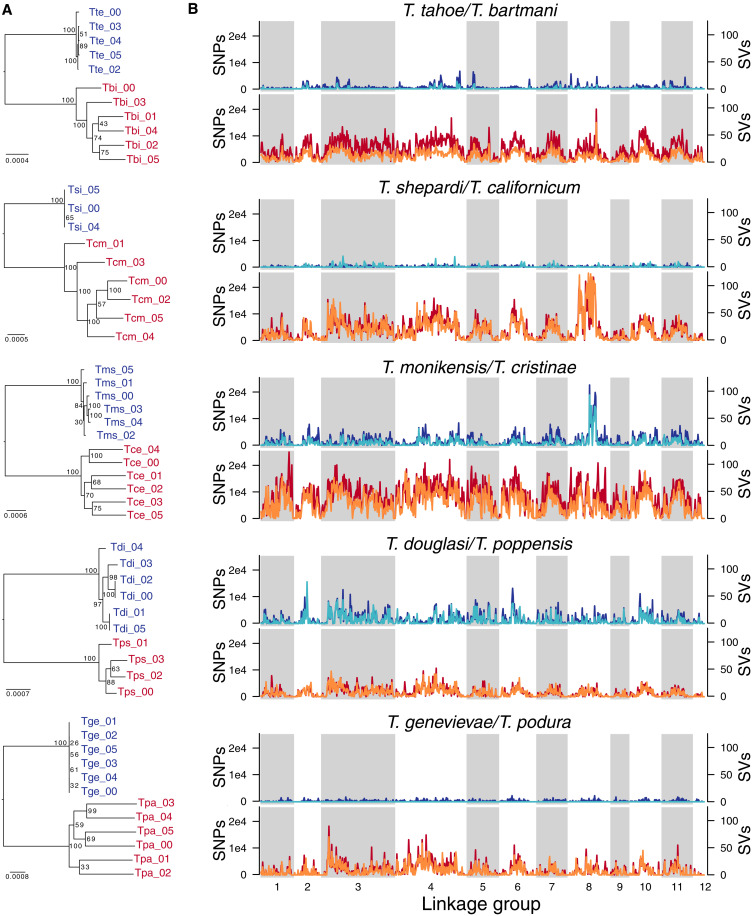
Population polymorphism levels in parthenogenetic (blue) and sexual (red) *Timema* species. (**A**) Phylogenies based on 1:1 orthologous genes reflect the different levels of genotype diversities in parthenogenetic *Timema* species. (**B**) Distribution of SVs (dark blue and red) and SNPs (light blue and orange) along the genome. Scaffolds from the 10 de novo genomes are anchored on autosomal linkage groups from the sexual species *T. cristinae* (see Supplementary Text).

To address these aspects in the genomes of sexual and parthenogenetic *Timema* species, we mapped population-level variation for the SNPs and SVs inferred above to our species-specific reference genomes. We then anchored our reference genome scaffolds to the 12 autosomal linkage groups of a previously published assembly of the sexual species *T. cristinae* [v1.3 from (*30*); see Supplementary Text]. This revealed that different types of polymorphisms (SNPs and SVs) tended to co-occur across the genomes in all species, independently of reproductive mode (Fig. 3).

The focal population for three of the five parthenogenetic species (*T. genevievae*, *T. tahoe*, and *T. shepardi*) consisted largely of a single genotype with only minor variation among individuals. By contrast, genotype diversity was considerable in *T. monikensis* and *T. douglasi* (Fig. 3A). In the former species, there was further a conspicuous diversity peak on LG8, supporting the idea that parthenogenesis is automictic in *Timema*. Under complete linkage (functionally mitotic parthenogenesis), putative effects of selection on this LG would be expected to propagate to the whole genome. Independently of local diversity peaks, overall diversity levels in *T. monikensis* and *T. douglasi* were comparable to the diversities in populations of some of the sexual *Timema* species (Fig. 3A). Different mechanisms could contribute to such unexpected diversities in parthenogenetic *Timema*, including the presence of lineages that derived independently from their sexual ancestor, or rare sex. While a single transition to parthenogenesis is believed to have occurred in *T. monikensis*, the nominal species *T. douglasi* is polyphyletic and known to consist of independently derived clonal lineages. These lineages have broadly different geographic distributions but can overlap locally (*31*). Identifying the causes of genotypic variation in these species, including the possibility of rare sex, requires further investigation and is a challenge for future studies.

Independently of the mechanisms underlying polymorphism in the parthenogenetic species *T. monikensis*, the polymorphism peak on LG8 is notable (Fig. 3B). This peak occurs in a region previously shown to determine color morph [green, green-striped, or brownish (“melanistic”)] in the sexual sister species of *T. monikensis*, *T. cristinae* (*30*). Our focal *T. monikensis* population features four discrete color morphs (green, dark brown, yellow, and beige), suggesting that additional color morphs may be regulated by the region identified in *T. cristinae*. We also found a peak in polymorphism on LG8, spanning over approximately two-thirds of LG8, in the sexual species *T. californicum*, which features a different panel of color morphs than *T. cristinae* (*32*). This diversity peak in *T. californicum* was generated by the presence of two divergent haplotypes (approximately 24 Mbp long), with gray individuals homozygous for one haplotype and green individuals heterozygous or homozygous for the alternative haplotype (see Supplementary Text). Note that the gray color morph is not known in the monomorphic green parthenogenetic sister of *T. californicum* (*T. shepardi*), and we therefore do not expect the same pattern of polymorphism on LG8 in this species.

### Faster rate of adaptive evolution in sexual than parthenogenetic species

We have shown previously that parthenogenetic *Timema* species accumulate deleterious mutations faster than sexual species (*33*, *34*), a pattern also reported in other parthenogenetic taxa [reviewed in (*16*, *35*)]. This is expected given that increased linkage among loci in parthenogens reduces the ability of selection to act individually on each locus, which generates different forms of selective interference (*9*, *10*, *36*). In addition to facilitating the accumulation of deleterious mutations, selective interference among loci in parthenogens should also constrain the efficiency of positive selection. While there is accumulating evidence for this process in experimental evolution studies [e.g., (*37*–*39*)], its impact on natural populations remains unclear (*16*, *35*). To compare the efficiency of positive selection in sexual and parthenogenetic *Timema*, we used a branch-site model on the gene trees [see (*40*) and Methods]. We compared the terminal branches leading to sexual or parthenogenetic species in one-to-one orthologous genes identified in at least three species pairs (data S1), using a threshold of *q* < 0.05 to classify which terminal branches show evidence of positive selection.

**Fig. 4.**
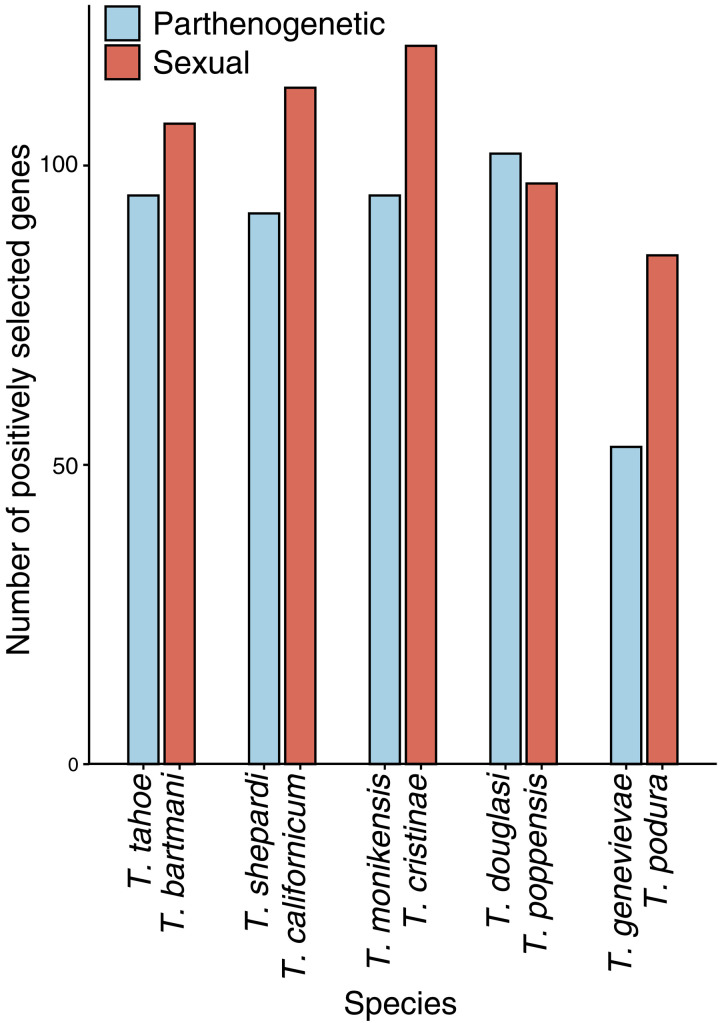
Number of genes showing evidence for positive selection in each species (total number of genes = 7155). In addition to reproductive mode, species pair also had a significant influence on the number of positively selected branches (binomial GLMM *P* = 0.015). There was no significant interaction between species pair and reproductive mode (*P* = 0.197). Note that the difference between reproductive modes is robust to a more stringent cutoff (*q* < 0.01 instead of 0.05; fig. S2A) and if genes with polymorphic positively selected sites were excluded (fig. S2B).

We found a greater number of positively selected genes in sexual than parthenogenetic species [binomial generalized linear mixed model (GLMM) *P* = 0.005; Fig. 4]. In addition, we also examined if there was more evidence for positive selection in sexual species in a threshold-free way by comparing the likelihood ratio test statistic between parthenogenetic and sexual species. This confirmed that the evidence for positive selection was stronger for sexual species (permutation glm *P* = 0.011).

The positively selected genes that we identified are most likely associated with species-specific adaptations. Few of them were shared between species, with overlap between species not greater than expected by chance (false discovery rate < 0.4; fig. S3), and there was little enrichment of functional processes in positively selected genes [0 to 19 Gene Ontology (GO) terms per species; table S5]. Most of the significant GO terms were associated with positively selected genes in parthenogenetic *Timema* (table S5), likely because a much smaller proportion of positively selected genes in sexual species had annotations (fig. S4). We speculate that positively selected genes in sexuals could often be involved in sexual selection and species recognition. Genes associated with processes such as pheromone production and reception often evolve very fast, which makes them difficult to annotate through homology-based inference (*41*). For the parthenogenetic species, although some terms could be associated with their mode of reproduction (e.g., GO:0033206 meiotic cytokinesis in *T. douglasi*), most are not clearly linked to a parthenogenetic life cycle.

### TE content is similar between species with sexual and parthenogenetic reproduction

Upon the loss of sexual reproduction, TE dynamics are expected to change (*14*, *42*). How these changes affect genome-wide TE loads is, however, unclear as sex can facilitate both the spread and the elimination of TEs (*16*). In parthenogens, TE load might initially increase as a result of weaker purifying selection, a pattern well illustrated by the accumulation of TEs in nonrecombining parts of sex chromosomes and other supergenes (*43*, *44*). However, TE loads in parthenogens are expected to decrease over time via at least two nonmutually exclusive mechanisms. First, TEs are expected to evolve lower activity over time as their evolutionary interests are aligned with their hosts (*14*, *42*). Second, TE copies that were purged via excision can recolonize a sexual but not a parthenogenetic genomic background (*15*). Last, it is important to note that the predicted effects of reproductive mode on genomic TE loads require sufficient levels of TE activity (transposition or excision). If TE activity is very low, TE contents will remain relatively stable in closely related species independently of their reproductive mode.

**Fig. 5.**
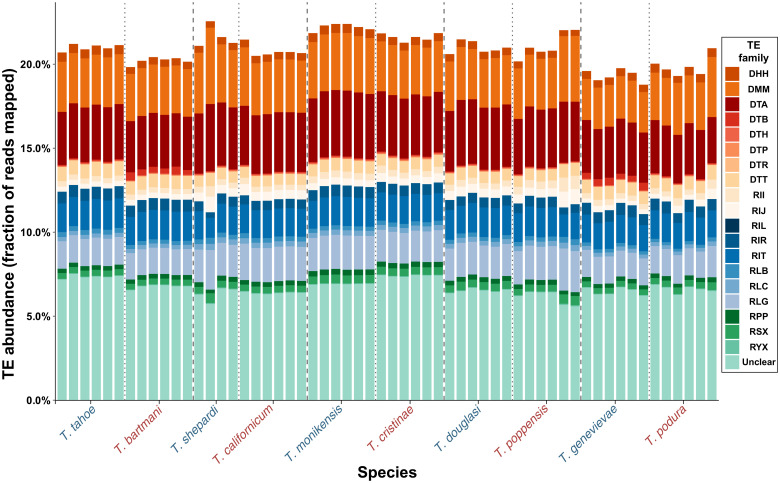
Total TE abundance in the 10 *Timema* species. TE abundance in each genome is expressed as the fraction of reads that map to a genus-level TE library (replicates within species correspond to the reference genome and three to five resequenced individuals). TE families are named following the Wicker classification (*65*). The first character corresponds to the TE class [class I are retrotransposons (R); class II are DNA transposons (D)], the second character corresponds to the order (e.g., LTR), and the third character corresponds to the superfamily (e.g., *Gypsy*); for example, RLG is a *Gypsy* retroelement. The character X indicates unknown classification at the superfamily level (because of fragmentation or lack of detectable homology).

We generated a *Timema* genus-level TE library by merging de novo TE libraries generated separately for each of the 10 *Timema* species. We then quantified TE content in each *Timema* genome by mapping reads to this merged library (see Methods). The overall TE content was very similar in all 10 species (20 to 23.6%), with significant differences in abundance of TE superfamilies between species groups but no significant effect of reproductive mode (*P* = 0.43; Fig. 5 and fig. S5).

The oldest node in our *Timema* phylogeny has an age estimate of 30 million years (*45*), but the overall TE contents of the two clades separating at this node only differ by 1.3%. This suggests that there has been little TE activity during the evolution of the genus or that transposition and excision rates are in balance. TE sequence divergence landscapes further revealed that there were few recently duplicated TEs in the *Timema* genomes, again suggesting little recent TE activity (see Supplementary Text and fig. S13). Although additional studies are required to formally distinguish between low TE activity and balanced transposition and excision rates, our results suggest that TE content likely evolves too slowly in *Timema* for any putative reproductive mode effects to become apparent. Low activity of TEs might facilitate the persistence of incipient parthenogenetic strains (*16*) and thus help to explain the high frequency of established parthenogenetic species in *Timema*.

In conclusion, we present genomes of five independently derived parthenogenetic lineages of *Timema* stick insects, together with their five sexual sister species. This design with replicated species pairs allows us to disentangle consequences of parthenogenesis from species-specific effects. All parthenogenetic *Timema* species are largely or completely homozygous for both SNPs and SVs, and frequently feature lower levels of population polymorphism than their close sexual relatives. Low population polymorphism can exacerbate the effects of linkage for reducing the efficacy of selection, resulting in reduced rates of positive selection in parthenogenetic *Timema*, in addition to the accumulation of deleterious mutations previously documented (*33*). Despite these negative genomic consequences, parthenogenesis is an unusually successful strategy in *Timema*. It evolved and persisted repeatedly in the genus, and parthenogenetic species often occur across large geographic areas. Because *Timema* are wingless and their populations are subjected to frequent extinction-recolonization dynamics in their fire-prone Californian shrubland habitats, the genomic costs of parthenogenesis are likely offset by one of the most classical benefits of parthenogenesis: the ability to reproduce without a mate.

## METHODS

### Sample collection and sequencing

For each of the 10 species, the DNA for Illumina shotgun sequencing was derived from virgin adult females collected in 2015 from natural populations in California (table S1). Extractions were done using the Qiagen MagAttract HMW DNA Kit, following the manufacturer’s indications. Five polymerase chain reaction (PCR)–free libraries were generated for each reference genome (three 2× 125-bp paired-end libraries with average insert sizes of 350, 550, and 700 bp, respectively, and two mate-pair libraries with 3000- and 5000-bp insert sizes, respectively); one library (550-bp insert size) was generated for each resequenced individual. Libraries were prepared using the Illumina TruSeq DNA PCR-Free or Nextera Mate Pair Library Prep Kits, following the manufacturer’s instructions, and sequenced on the Illumina HiSeq 2500 system, using v4 chemistry and 2× 125-bp reads at FASTERIS SA, Plan-les-Ouates, Switzerland and the Lausanne Genomic Technologies Facility, Switzerland.

### Genome assembly and annotation

The total coverage for the reference genomes (all libraries combined) ranged between 37× and 45× (table S2). Trimmed paired-end reads were assembled into contigs using ABySS (*46*) and further scaffolded using paired-end and mate pairs using BESST (*47*). Scaffolds identified as contaminants were filtered using BlobTools (*48*). The assembly details can be found in the Supplementary Materials (see Supplementary Text).

Publicly available RNA sequencing libraries for *Timema* (*33*, *49*, *50*) were used as expression evidence for annotation. Trimmed reads were assembled using Trinity v2.5.1 (*51*) to produce reference-guided transcriptomes. The transcriptomes and protein evidence were combined with ab initio gene finders to predict protein coding genes using MAKER v2.31.8 (*52*). The annotation details can be found in the Supplementary Materials (see Supplementary Text).

### Orthologs

*Timema* orthologous groups (OGs) were inferred with the OrthoDB standalone pipeline (v. 2.4.4) using default parameters (*53*). In short, genes are clustered with a graph-based approach based on all best reciprocal hits between each pair of genomes. The high level of fragmentation typical for Illumina-based genomes constrains the ability to identify 1:1 orthologs across all 10 *Timema* species. To maximize the number of single-copy OGs covering all 10 *Timema* species, transcriptomes were included during orthology inference. Thus, transcripts were used to complete OGs in the absence of a gene from the corresponding species. Using this approach, 7157 single-copy OGs covering at least three sexual-parthenogenetic sister species pairs were obtained (data S1).

### Horizontal gene transfers

To detect HGT from nonmetazoan species, we first used the pipeline of foreign sequence detection developed by Francois *et al*. (*54*). We used the set of coding DNA sequences (CDS) identified in publicly available transcriptomes (*33*) and the genome assemblies before the decontamination procedure with BlobTools (*48*). The rationale is that some genuine HGT could have been wrongly considered as contaminant sequences during this decontamination step and thus been removed from the assembly. Scaffolds filtered during decontamination are available from our github repository (https://github.com/AsexGenomeEvol/Timema_asex_genomes/tree/main/4_Horizontal_Gene_Transfers/contamination_sequences) and will be archived upon acceptance.

Briefly, a DIAMOND BlastP (v0.8.33) (*55*) allows us to detect candidate nonmetazoan genes in the set of CDS of each species. Taxonomic assignment is based on the 10 best blast hits to account for potential contaminations and other sources of taxonomic misassignment using a curated reference database designed to cover all domains of life [see Francois *et al*. (*54*) for details]. Candidate nonmetazoan sequences are then subjected to a synteny-based screen with Gmap (v2016-11-07) (*56*) to discriminate between contaminant sequences and potential HGT-derived sequences. A sequence is considered as an HGT candidate if it is physically linked to (i.e., mapped to the same scaffold as) at least one “confident-arthropod” CDS (previously identified in the DIAMOND blast).

We then clustered all HGT candidates identified in each of the 10 *Timema* species into HGT families using Silix (v1.2.10) (*57*), requiring a minimum of 85% identity (default parameters otherwise). These HGT families were then “completed” as much as possible by adding homologs from the genome assemblies not identified as HGT candidates (this could occur if the corresponding sequences are fragmented or on short scaffolds for example). To this end, the longest sequence of each HGT family was mapped (using Gmap) on the genomic scaffolds of all species, requiring a minimum of 85% identity.

For each completed HGT family, a protein alignment of the candidate HGT sequence(s) and its (their) 50 best DIAMOND blastP hits in the reference database (first step of the pipeline) was generated with MAFFT (v7) (*58*). The alignments were cleaned using HMMcleaner (stringency parameter = 12) (*59*), and sites with more than 50% missing data were removed. Phylogenetic trees were inferred using RAxML (v8.2) (*60*) with the model “PROTGAMMALGX” of amino acid substitution and 100 bootstrap replicates. Phylogenetic trees were inspected by eye to confirm whether an evolutionary history was consistent with the hypothesis of HGT.

### Heterozygosity

Genome-wide nucleotide heterozygosity was estimated using genome profiling analysis of raw reads from the reference genomes using GenomeScope (v2) (*20*). The second SNP-based heterozygosity estimate was generated using resequenced individuals. We resequenced five individuals per species, but three individuals of *T. shepardi*, two individuals of *T. poppensis*, and one *T. tahoe* individual did not pass quality control and were discarded from all downstream analyses. SNP calling was based on the Genome Analysis Toolkit best practices pipeline (*61*). We used a conservative set of SNPs with quality scores ≥300 and supported by 15× coverage in at least one of the individuals. SNP heterozygosity was then estimated as the number of heterozygous SNPs divided by the number of callable sites in each genome. Because of stringent filtering criteria, our SNP-based heterozygosity is an underestimation of genome-wide heterozygosity.

### Structural variants

We used Manta (v1.5.0) (*62*), a diploid-aware pipeline for SV calling, in the same set of resequenced individuals used for SNP heterozygosity estimates. We found a high frequency of heterozygous SVs with approximately twice the expected coverage (fig. S6), which likely represent false positives. To reduce the number of false positives, we filtered very short SVs (30 bases or less) and kept only variant calls that had either split read or paired-end read support within the expected coverage range, where the coverage range was defined individually for each sample by manual inspection of coverage distributions. The filtered SV calls were subsequently merged into population SV calls using SURVIVOR (v1.0.2) (*63*). The merging criteria were SV calls of the same type on the same strand with breakpoint distances shorter than 100 bp.

### Genome alignment

We anchored our genome assemblies to the reference of *T. cristinae* (BioProject Accession PRJNA417530) (*30*) using MUMmer (version 4.0.0beta2) (*64*) with parameter --mum. The alignments were processed by other tools within the package: show-coords with parameters -THrcl to generate tab-delimited alignment files and dnadiff to generate one-to-one alignments. We used only uniquely anchored scaffolds for which we were able to map at least 10,000 nucleotides to the *T. cristinae* reference genome.

### Transposable elements

For each species, specific repeat libraries were constructed and annotated to the TE superfamily level (*65*), wherever possible. For collecting repetitive sequences, we used a raw read-based approach DNAPipeTE v1.2 (*66*) with parameters -genome_coverage 0.5 -sample_number 4 and respective species genome size, as well as an assembly-based approach (RepeatModeler v1.0.8 available at www.repeatmasker.org/RepeatModeler/), such that repeats not present in the assembly can still be represented in the repeat library. The two raw libraries were merged and clustered by 95% identity (the TE family threshold) using usearch v10.0.240 (*67*) with the centroid option. To annotate TEs larger than 500 bp in the repeat library, we used an approach that combines homology and structural evidence [PASTEClassifier (*68*)]. Because PASTEClassifier did not annotate to TE superfamily levels, we additionally compared by BlastN (v. 2.7.1+) (*69*) the repeat libraries to the well curated *T. cristinae* TE library from Soria-Carrasco *et al*. (*18*). Blast hits were filtered according to TE classification standards: identity percentage >80%, alignment length >80 bp, and the best hit per contig was kept. The two classification outputs were compared, and in case of conflict, the classification level of PASTEClassifier was preferred. All nonannotated repeats were labeled “unknown.” Repeat library header naming was done according to RepeatMasker standard, but keeping the Wicker naming for elements (i.e., Wicker#Repeatmasker, e.g., DTA#DNA/hAT). TE libraries were sorted by header, and TE annotations to similar families were numbered consecutively. Species-specific TE libraries were merged into a genus-level *Timema* TE library to account for any TE families that might have not been detected in the single species assemblies.

To estimate the TE content of reference genomes and resequenced individuals, we first repeat-masked the assemblies with the genus-level TE library using RepeatMasker v4.1.0 with parameters set as -gccalc -gff -u -a -xsmall -no_is -div 30 -engine rmblast (*70*). Second, we mapped the 350-bp insert paired-end reads back to the reference genome assemblies using BWA-MEM v0.7.17 (*71*) with standard parameters. We then counted the fraction of reads mapping to TEs out of total mappable reads by counting the number of reads that mapped to each genomic location annotated as TE using htseq-counts (v0.6.1.1p1) (*72*) with parameters set to -r name -s no -t similarity -i Target --nonunique none, using the mapped read alignments and the gff output of RepeatMasker (filtered for TE length of >80 bp). TE content was compared among species using a permutation ANOVA with 5000 bootstrap replicates.

To generate the TE activity landscapes, utilities scripts calcDivergenceFromAlign.pl and createRepeatLandscape_mod.pl were used on the outputs of Repeatmasker v4.1.0. The createRepeatLandscape_mod.pl was modified to match the TE families found in *Timema*.

### Positive selection analysis

Only one-to-one orthologs in at least three pairs of species (sister-species sex-asex) were used. The species phylogeny was imposed on every gene as the “gene tree.” We used a customized version of the Selectome pipeline (*73*). All alignment building and filtering was performed on predicted amino acid sequences, and the final amino acid MSAs (multiple sequence alignments) were used to infer the nucleotide MSAs used for positive selection inference. MSAs were obtained by MAFFT (v. 7.310) (*58*) with the allowshift option, which avoids overaligning nonhomologous regions (e.g., gene prediction errors, or alternative transcripts). All the next steps “mask” rather than remove sites, by replacing the amino acid with an “X” and the corresponding codon with “NNN.” MCoffee (v11.00.8cbe486) (*74*) was run with the following aligners: mafft_msa, muscle_msa, clustalo_msa (*75*), and t_coffee_msa (*76*). MCoffee provides a consistency score per amino acid, indicating how robust the alignment is at that position for that sequence. Residues with a consistency score less than five were masked. TrimAl (v. 1.4.1) (*77*) was used to mask columns with less than four residues (neither gap nor X). Following this step, 2 of the 7157 ortholog alignments consisted only of gaps and were excluded from further analyses.

The branch-site model with rate variation at the DNA level (*40*) was run using the Godon software (https://bitbucket.org/Davydov/godon/, version 2020-02-17, option BSG --ncat 4). Each branch was tested iteratively, in one run per gene tree. For each branch, we obtain a ΔlnL that measures the evidence for positive selection, a corresponding *P* value and associated *q* value (estimated from the distribution of *P* values over all branches of all genes), and an estimate of the proportion of sites under positive selection, if any. All positive selection results, and detailed methods, are available at https://selectome.org/timema. To determine whether the number of positively selected genes differed between sexual and parthenogenetic species, we used a binomial GLMM approach [lme4 (*78*)] with *q* value threshold of 0.05 or 0.01. Significance of model terms was determined with a Wald statistic. In addition, we also examined if there was more evidence for positive selection in sexual species in a threshold-free way by comparing ΔlnL values between parthenogenetic and sexual species. To do this, we used a permutation glm approach where reproductive mode (sexual or parthenogenetic) was randomly switched within a species pair. To determine whether the overlap of positively selected genes was greater than expected by chance, we used the SuperExactTest package (v. 0.99.4) (*79*) in R. The resulting *P* values were multiple test–corrected using Benjamini and Hochberg’s algorithm implemented in R. To assess the impact of polymorphism on our results, we repeated our analysis after excluding positively selected genes with nonsynonymous polymorphic variants at positively selected sites (those with a posterior probability of >0.95 detected by Bayes empirical Bayes analysis in Godon). Functional enrichment analyses were performed using TopGO (v. 2.28.0) (*80*) using the *Drosophila melanogaster* functional annotation (see Supplementary Text). To determine whether a GO term was enriched, we used a Fisher’s exact test with the “weight01” algorithm to account for the GO topology. GO terms were considered to be significantly enriched when *P* < 0.05.

## Supplementary Materials

### This PDF file includes:

Supplementary Text Figs. S1 to S14 Tables S1 to S9 References

## Notes

View/request a protocol for this paper from *Bio-protocol*.

## References

[1] M. Neiman, C. M. Lively, S. Meirmans, Why sex? A pluralist approach revisited. Trends Ecol. Evol. 32, 589–600 (2017). 28606425 10.1016/j.tree.2017.05.004

[2] N. P. Sharp, S. P. Otto, Evolution of sex: Using experimental genomics to select among competing theories. Bioessays 38, 751–757 (2016). 27315146 10.1002/bies.201600074

[3] G. Bell, *The Masterpiece of Nature: The Evolution and Genetics of Sexuality* (University of California Press, 1982).

[4] G. C. Williams, *Sex and Evolution* (Princeton Univ. Press, 1975).

[5] J. Maynard Smith, *The Evolution of Sex* (Cambridge Univ. Press, 1978).

[6] J. Gerritsen, Sex and parthenogenesis in sparse populations. Am. Nat. 115, 718–742 (1980).

[7] S. K. Jain, The evolution of inbreeding in plants. Annu. Rev. Ecol. Syst. 7, 469–495 (1976).

[8] J. Felsenstein, The evolutionary advantage of recombination. Genetics 78, 737–756 (1974). 4448362 10.1093/genetics/78.2.737 PMC1213231

[9] W. G. Hill, A. Robertson, The effect of linkage on limits to artificial selection. Genet. Res. 8, 269–294 (1966). 5980116

[10] P. D. Keightley, S. P. Otto, Interference among deleterious mutations favours sex and recombination in finite populations. Nature 443, 89–92 (2006). 16957730 10.1038/nature05049

[11] N. H. Barton, Why sex and recombination? Cold Spring Harb. Symp. Quant. Biol. 74, 187–195 (2009). 19903748 10.1101/sqb.2009.74.030

[12] C. W. Birky Jr., Heterozygosity, heteromorphy, and phylogenetic trees in asexual eukaryotes. Genetics 144, 427–437 (1996). 8878706 10.1093/genetics/144.1.427 PMC1207515

[13] D. Mark Welch, M. Meselson, Evidence for the evolution of bdelloid rotifers without sexual reproduction or genetic exchange. Science 288, 1211–1215 (2000). 10817991 10.1126/science.288.5469.1211

[14] D. A. Hickey, Selfish DNA: A sexually-transmitted nuclear parasite. Genetics 101, 519–531 (1982). 6293914 10.1093/genetics/101.3-4.519 PMC1201875

[15] E. S. Dolgin, B. Charlesworth, The effects of recombination rate on the distribution and abundance of transposable elements. Genetics 178, 2169–2177 (2008). 18430942 10.1534/genetics.107.082743 PMC2323806

[16] K. S. Jaron, J. Bast, R. W. Nowell, T. R. Ranallo-Benavidez, M. Robinson-Rechavi, T. Schwander, Genomic features of parthenogenetic animals. J. Hered. 112, 19–33 (2021). 32985658 10.1093/jhered/esaa031 PMC7953838

[17] T. Schwander, B. J. Crespi, Multiple direct transitions from sexual reproduction to apomictic parthenogenesis in *Timema* stick insects. Evolution 63, 84–103 (2009). 18803687 10.1111/j.1558-5646.2008.00524.x

[18] V. Soria-Carrasco, Z. Gompert, A. A. Comeault, T. E. Farkas, T. L. Parchman, J. S. Johnston, C. A. Buerkle, J. L. Feder, J. Bast, T. Schwander, S. P. Egan, B. J. Crespi, P. Nosil, Stick insect genomes reveal natural selection’s role in parallel speciation. Science 344, 738–742 (2014). 24833390 10.1126/science.1252136

[19] R. M. Waterhouse, M. Seppey, F. A. Simão, M. Manni, P. Ioannidis, G. Klioutchnikov, E. V. Kriventseva, E. M. Zdobnov, BUSCO applications from quality assessments to gene prediction and phylogenomics. Mol. Biol. Evol. 35, 543–548 (2018). 29220515 10.1093/molbev/msx319 PMC5850278

[20] T. R. Ranallo-Benavidez, K. S. Jaron, M. C. Schatz, GenomeScope 2.0 and Smudgeplot for reference-free profiling of polyploid genomes. Nat. Commun. 11, 1432 (2020). 32188846 10.1038/s41467-020-14998-3 PMC7080791

[21] J. Romiguier, P. Gayral, M. Ballenghien, A. Bernard, V. Cahais, A. Chenuil, Y. Chiari, R. Dernat, L. Duret, N. Faivre, E. Loire, J. M. Lourenco, B. Nabholz, C. Roux, G. Tsagkogeorga, A. A.-T. Weber, L. A. Weinert, K. Belkhir, N. Bierne, S. Glémin, N. Galtier, Comparative population genomics in animals uncovers the determinants of genetic diversity. Nature 515, 261–263 (2014). 25141177 10.1038/nature13685

[22] T. Schwander, S. Vuilleumier, J. Dubman, B. J. Crespi, Positive feedback in the transition from sexual reproduction to parthenogenesis. Proc. Biol. Sci. 277, 1435–1442 (2010). 20071382 10.1098/rspb.2009.2113 PMC2871946

[23] J. Engelstädter, Asexual but not clonal: Evolutionary processes in automictic populations. Genetics 206, 993–1009 (2017). 28381586 10.1534/genetics.116.196873 PMC5499200

[24] M. Neiman, T. Schwander, Using parthenogenetic lineages to identify advantages of sex. Evol. Biol. 38, 115–123 (2011).

[25] S. Glémin, C. M. François, N. Galtier, Genome evolution in outcrossing vs. selfing vs. asexual species. Methods Mol. Biol. 1910, 331–369 (2019). 31278670 10.1007/978-1-4939-9074-0_11

[26] M. Pearcy, S. Aron, C. Doums, L. Keller, Conditional use of sex and parthenogenesis for worker and queen production in ants. Science 306, 1780–1783 (2004). 15576621 10.1126/science.1105453

[27] M. O. Lorenzo-Carballa, A. Cordero-Rivera, Thelytokous parthenogenesis in the damselfly Ischnura hastata (Odonata, Coenagrionidae): Genetic mechanisms and lack of bacterial infection. Heredity 103, 377–384 (2009). 19513091 10.1038/hdy.2009.65

[28] T. J. Treangen, S. L. Salzberg, Repetitive DNA and next-generation sequencing: Computational challenges and solutions. Nat. Rev. Genet. 13, 36–46 (2011). 22124482 10.1038/nrg3117 PMC3324860

[29] H. Ellegren, N. Galtier, Determinants of genetic diversity. Nat. Rev. Genet. 17, 422–433 (2016). 27265362 10.1038/nrg.2016.58

[30] P. Nosil, R. Villoutreix, C. F. de Carvalho, T. E. Farkas, V. Soria-Carrasco, J. L. Feder, B. J. Crespi, Z. Gompert, Natural selection and the predictability of evolution in *Timema* stick insects. Science 359, 765–770 (2018). 29449486 10.1126/science.aap9125

[31] T. Schwander, L. Henry, B. J. Crespi, Molecular evidence for ancient asexuality in *Timema* stick insects. Curr. Biol. 21, 1129–1134 (2011). 21683598 10.1016/j.cub.2011.05.026

[32] V. R. Vickery, Revision *of Timema* scudder (Phasmatoptera: Timematodea) including three new species. Can. Entomol. 125, 657–692 (1993).

[33] J. Bast, D. J. Parker, Z. Dumas, K. M. Jalvingh, P. Tran Van, K. S. Jaron, E. Figuet, A. Brandt, N. Galtier, T. Schwander, Consequences of asexuality in natural populations: Insights from stick insects. Mol. Biol. Evol. 35, 1668–1677 (2018). 29659991 10.1093/molbev/msy058 PMC5995167

[34] L. Henry, T. Schwander, B. J. Crespi, Deleterious mutation accumulation in asexual Timema stick insects. Mol. Biol. Evol. 29, 401–408 (2012). 21940645 10.1093/molbev/msr237

[35] M. Neiman, P. G. Meirmans, T. Schwander, S. Meirmans, Sex in the wild: How and why field-based studies contribute to solving the problem of sex. Evolution 72, 1194–1203 (2018). 29645091 10.1111/evo.13485

[36] S. P. Otto, Selective interference and the evolution of sex. J. Hered. 112, 9–18 (2020). 10.1093/jhered/esaa026 33047117

[37] M. J. McDonald, D. P. Rice, M. M. Desai, Sex speeds adaptation by altering the dynamics of molecular evolution. Nature 531, 233–236 (2016). 26909573 10.1038/nature17143 PMC4855304

[38] O. Kaltz, G. Bell, The ecology and genetics of fitness in Chlamydomonas. XII. Repeated sexual episodes increase rates of adaptation to novel environments. Evolution 56, 1743–1753 (2002). 12389719 10.1111/j.0014-3820.2002.tb00188.x

[39] M. R. Goddard, H. C. J. Godfray, A. Burt, Sex increases the efficacy of natural selection in experimental yeast populations. Nature 434, 636–640 (2005). 15800622 10.1038/nature03405

[40] I. I. Davydov, N. Salamin, M. Robinson-Rechavi, Large-scale comparative analysis of codon models accounting for protein and nucleotide selection. Mol. Biol. Evol. 36, 1316–1332 (2019). 30847475 10.1093/molbev/msz048 PMC6526913

[41] W. Haerty, S. Jagadeeshan, R. J. Kulathinal, A. Wong, K. Ravi Ram, L. K. Sirot, L. Levesque, C. G. Artieri, M. F. Wolfner, A. Civetta, R. S. Singh, Evolution in the fast lane: Rapidly evolving sex-related genes in Drosophila. Genetics 177, 1321–1335 (2007). 18039869 10.1534/genetics.107.078865 PMC2147986

[42] B. Charlesworth, C. H. Langley, The evolution of self-regulated transposition of transposable elements. Genetics 112, 359–383 (1986). 3000868 10.1093/genetics/112.2.359 PMC1202706

[43] T. Schwander, R. Libbrecht, L. Keller, Supergenes and complex phenotypes. Curr. Biol. 24, R288–R294 (2014). 24698381 10.1016/j.cub.2014.01.056

[44] D. Bachtrog, Y-chromosome evolution: Emerging insights into processes of Y-chromosome degeneration. Nat. Rev. Genet. 14, 113–124 (2013). 23329112 10.1038/nrg3366 PMC4120474

[45] R. Riesch, M. Muschick, D. Lindtke, R. Villoutreix, A. A. Comeault, T. E. Farkas, K. Lucek, E. Hellen, V. Soria-Carrasco, S. R. Dennis, C. F. de Carvalho, R. J. Safran, C. P. Sandoval, J. Feder, R. Gries, B. J. Crespi, G. Gries, Z. Gompert, P. Nosil, Transitions between phases of genomic differentiation during stick-insect speciation. Nat. Ecol. Evol. 1, 0082 (2017). 10.1038/s41559-017-0082 28812654

[46] S. D. Jackman, B. P. Vandervalk, H. Mohamadi, J. Chu, S. Yeo, S. A. Hammond, G. Jahesh, H. Khan, L. Coombe, R. L. Warren, I. Birol, ABySS 2.0: Resource-efficient assembly of large genomes using a Bloom filter. Genome Res. 27, 768–777 (2017). 28232478 10.1101/gr.214346.116 PMC5411771

[47] K. Sahlin, R. Chikhi, L. Arvestad, Assembly scaffolding with PE-contaminated mate-pair libraries. Bioinformatics 32, 1925–1932 (2016). 27153683 10.1093/bioinformatics/btw064

[48] D. R. Laetsch, M. L. Blaxter, BlobTools: Interrogation of genome assemblies. F1000Res. 6, 1287 (2017).

[49] D. J. Parker, J. Bast, K. Jalvingh, Z. Dumas, M. Robinson-Rechavi, T. Schwander, Sex-biased gene expression is repeatedly masculinized in asexual females. Nat. Commun. 10, 4638 (2019). 31604947 10.1038/s41467-019-12659-8 PMC6789136

[50] D. J. Parker, J. Bast, K. Jalvingh, Z. Dumas, M. Robinson-Rechavi, T. Schwander, Repeated evolution of asexuality involves convergent gene expression changes. Mol. Biol. Evol. 36, 350–364 (2019). 30445505 10.1093/molbev/msy217 PMC6404633

[51] B. J. Haas, A. Papanicolaou, M. Yassour, M. Grabherr, P. D. Blood, J. Bowden, M. B. Couger, D. Eccles, B. Li, M. Lieber, M. D. MacManes, M. Ott, J. Orvis, N. Pochet, F. Strozzi, N. Weeks, R. Westerman, T. William, C. N. Dewey, R. Henschel, R. D. LeDuc, N. Friedman, A. Regev, De novo transcript sequence reconstruction from RNA-seq using the Trinity platform for reference generation and analysis. Nat. Protoc. 8, 1494–1512 (2013). 23845962 10.1038/nprot.2013.084 PMC3875132

[52] C. Holt, M. Yandell, MAKER2: An annotation pipeline and genome-database management tool for second-generation genome projects. BMC Bioinformatics 12, 491 (2011). 22192575 10.1186/1471-2105-12-491 PMC3280279

[53] E. V. Kriventseva, F. Tegenfeldt, T. J. Petty, R. M. Waterhouse, F. A. Simão, I. A. Pozdnyakov, P. Ioannidis, E. M. Zdobnov, OrthoDB v8: Update of the hierarchical catalog of orthologs and the underlying free software. Nucleic Acids Res. 43, D250–D256 (2015). 25428351 10.1093/nar/gku1220 PMC4383991

[54] C. M. Francois, F. Durand, E. Figuet, N. Galtier, Prevalence and implications of contamination in public genomic resources: A case study of 43 reference arthropod assemblies. G3 10, 721–730 (2020). 31862787 10.1534/g3.119.400758 PMC7003083

[55] B. Buchfink, C. Xie, D. H. Huson, Fast and sensitive protein alignment using DIAMOND. Nat. Methods 12, 59–60 (2015). 25402007 10.1038/nmeth.3176

[56] T. D. Wu, C. K. Watanabe, GMAP: A genomic mapping and alignment program for mRNA and EST sequences. Bioinformatics 21, 1859–1875 (2005). 15728110 10.1093/bioinformatics/bti310

[57] V. Miele, S. Penel, L. Duret, Ultra-fast sequence clustering from similarity networks with SiLiX. BMC Bioinformatics 12, 116 (2011). 21513511 10.1186/1471-2105-12-116 PMC3095554

[58] K. Katoh, D. M. Standley, MAFFT multiple sequence alignment software version 7: Improvements in performance and usability. Mol. Biol. Evol. 30, 772–780 (2013). 23329690 10.1093/molbev/mst010 PMC3603318

[59] A. Di Franco, R. Poujol, D. Baurain, H. Philippe, Evaluating the usefulness of alignment filtering methods to reduce the impact of errors on evolutionary inferences. BMC Evol. Biol. 19, 21 (2019). 30634908 10.1186/s12862-019-1350-2 PMC6330419

[60] A. Stamatakis, RAxML version 8: A tool for phylogenetic analysis and post-analysis of large phylogenies. Bioinformatics 30, 1312–1313 (2014). 24451623 10.1093/bioinformatics/btu033 PMC3998144

[61] G. A. Van der Auwera, M. O. Carneiro, C. Hartl, R. Poplin, G. Del Angel, A. Levy-Moonshine, T. Jordan, K. Shakir, D. Roazen, J. Thibault, E. Banks, K. V. Garimella, D. Altshuler, S. Gabriel, M. A. DePristo, From FastQ data to high confidence variant calls: The Genome Analysis Toolkit best practices pipeline. Curr. Protoc. Bioinformatics 43, 11.10.1–11.10.33 (2013). 10.1002/0471250953.bi1110s43 PMC4243306 25431634

[62] X. Chen, O. Schulz-Trieglaff, R. Shaw, B. Barnes, F. Schlesinger, M. Källberg, A. J. Cox, S. Kruglyak, C. T. Saunders, Manta: Rapid detection of structural variants and indels for germline and cancer sequencing applications. Bioinformatics 32, 1220–1222 (2016). 26647377 10.1093/bioinformatics/btv710

[63] D. C. Jeffares, C. Jolly, M. Hoti, D. Speed, L. Shaw, C. Rallis, F. Balloux, C. Dessimoz, J. Bähler, F. J. Sedlazeck, Transient structural variations have strong effects on quantitative traits and reproductive isolation in fission yeast. Nat. Commun. 8, 14061 (2017). 28117401 10.1038/ncomms14061 PMC5286201

[64] S. Kurtz, A. Phillippy, A. L. Delcher, M. Smoot, M. Shumway, C. Antonescu, S. L. Salzberg, Versatile and open software for comparing large genomes. Genome Biol. 5, R12 (2004). 14759262 10.1186/gb-2004-5-2-r12 PMC395750

[65] T. Wicker, F. Sabot, A. Hua-Van, J. L. Bennetzen, P. Capy, B. Chalhoub, A. Flavell, P. Leroy, M. Morgante, O. Panaud, E. Paux, P. SanMiguel, A. H. Schulman, A unified classification system for eukaryotic transposable elements. Nat. Rev. Genet. 8, 973–982 (2007). 17984973 10.1038/nrg2165

[66] C. Goubert, L. Modolo, C. Vieira, C. ValienteMoro, P. Mavingui, M. Boulesteix, De novo assembly and annotation of the Asian tiger mosquito (Aedes albopictus) repeatome with dnaPipeTE from raw genomic reads and comparative analysis with the yellow fever mosquito (Aedes aegypti). Genome Biol. Evol. 7, 1192–1205 (2015). 25767248 10.1093/gbe/evv050 PMC4419797

[67] R. C. Edgar, Search and clustering orders of magnitude faster than BLAST. Bioinformatics 26, 2460–2461 (2010). 20709691 10.1093/bioinformatics/btq461

[68] C. Hoede, S. Arnoux, M. Moisset, T. Chaumier, O. Inizan, V. Jamilloux, H. Quesneville, PASTEC: An automatic transposable element classification tool. PLOS ONE 9, e91929 (2014). 24786468 10.1371/journal.pone.0091929 PMC4008368

[69] S. F. Altschul, T. L. Madden, A. A. Schäffer, J. Zhang, Z. Zhang, W. Miller, D. J. Lipman, Gapped BLAST and PSI-BLAST: A new generation of protein database search programs. Nucleic Acids Res. 25, 3389–3402 (1997). 9254694 10.1093/nar/25.17.3389 PMC146917

[70] A. Smit, R. Hubley, P. Green, RepeatMasker Open-4.0 (2013–2015); www.repeatmasker.org.

[71] H. Li, Aligning sequence reads, clone sequences and assembly contigs with BWA-MEM. arXiv:1303.3997 [q-bio.GN] (16 March 2013).

[72] S. Anders, P. T. Pyl, W. Huber, HTSeq—A Python framework to work with high-throughput sequencing data. Bioinformatics 31, 166–169 (2015). 25260700 10.1093/bioinformatics/btu638 PMC4287950

[73] S. Moretti, B. Laurenczy, W. H. Gharib, B. Castella, A. Kuzniar, H. Schabauer, R. A. Studer, M. Valle, N. Salamin, H. Stockinger, M. Robinson-Rechavi, Selectome update: Quality control and computational improvements to a database of positive selection. Nucleic Acids Res. 42, D917–D921 (2014). 24225318 10.1093/nar/gkt1065 PMC3964977

[74] I. M. Wallace, O. O’Sullivan, D. G. Higgins, C. Notredame, M-Coffee: Combining multiple sequence alignment methods with T-Coffee. Nucleic Acids Res. 34, 1692–1699 (2006). 16556910 10.1093/nar/gkl091 PMC1410914

[75] F. Sievers, A. Wilm, D. Dineen, T. J. Gibson, K. Karplus, W. Li, R. Lopez, H. McWilliam, M. Remmert, J. Söding, J. D. Thompson, D. G. Higgins, Fast, scalable generation of high-quality protein multiple sequence alignments using Clustal Omega. Mol. Syst. Biol. 7, 539 (2011). 21988835 10.1038/msb.2011.75 PMC3261699

[76] C. Notredame, D. G. Higgins, J. Heringa, T-Coffee: A novel method for fast and accurate multiple sequence alignment. J. Mol. Biol. 302, 205–217 (2000). 10964570 10.1006/jmbi.2000.4042

[77] S. Capella-Gutiérrez, J. M. Silla-Martínez, T. Gabaldón, trimAl: A tool for automated alignment trimming in large-scale phylogenetic analyses. Bioinformatics 25, 1972–1973 (2009). 19505945 10.1093/bioinformatics/btp348 PMC2712344

[78] D. Bates, M. Mächler, B. Bolker, S. Walker, Fitting linear mixed-effects models using lme4. J. Stat. Softw. 67, 1–48 (2015).

[79] M. Wang, Y. Zhao, B. Zhang, Efficient test and visualization of multi-set intersections. Sci. Rep. 5, 16923 (2015). 26603754 10.1038/srep16923 PMC4658477

[80] A. Alexa, J. Rahnenführer, T. Lengauer, Improved scoring of functional groups from gene expression data by decorrelating GO graph structure. Bioinformatics 22, 1600–1607 (2006). 16606683 10.1093/bioinformatics/btl140

[81] A. M. Bolger, M. Lohse, B. Usadel, Trimmomatic: A flexible trimmer for Illumina sequence data. Bioinformatics 30, 2114–2120 (2014). 24695404 10.1093/bioinformatics/btu170 PMC4103590

[82] J. O’Connell, O. Schulz-Trieglaff, E. Carlson, M. M. Hims, N. A. Gormley, A. J. Cox, NxTrim: Optimized trimming of Illumina mate pair reads. Bioinformatics 31, 2035–2037 (2015). 25661542 10.1093/bioinformatics/btv057

[83] J. T. Simpson, K. Wong, S. D. Jackman, J. E. Schein, S. J. M. Jones, I. Birol, ABySS: A parallel assembler for short read sequence data. Genome Res. 19, 1117–1123 (2009). 19251739 10.1101/gr.089532.108 PMC2694472

[84] R. Chikhi, P. Medvedev, Informed and automated k-mer size selection for genome assembly. Bioinformatics 30, 31–37 (2014). 23732276 10.1093/bioinformatics/btt310

[85] R. Luo, B. Liu, Y. Xie, Z. Li, W. Huang, J. Yuan, G. He, Y. Chen, Q. Pan, Y. Liu, J. Tang, G. Wu, H. Zhang, Y. Shi, Y. Liu, C. Yu, B. Wang, Y. Lu, C. Han, D. W. Cheung, S.-M. Yiu, S. Peng, Z. Xiaoqian, G. Liu, X. Liao, Y. Li, H. Yang, J. Wang, T.-W. Lam, J. Wang, SOAPdenovo2: An empirically improved memory-efficient short-read de novo assembler. Gigascience 1, 18 (2012). 23587118 10.1186/2047-217X-1-18 PMC3626529

[86] E. W. Sayers, J. Beck, J. R. Brister, E. E. Bolton, K. Canese, D. C. Comeau, K. Funk, A. Ketter, S. Kim, A. Kimchi, P. A. Kitts, A. Kuznetsov, S. Lathrop, Z. Lu, K. McGarvey, T. L. Madden, T. D. Murphy, N. O’Leary, L. Phan, V. A. Schneider, F. Thibaud-Nissen, B. W. Trawick, K. D. Pruitt, J. Ostell, Database resources of the National Center for Biotechnology Information. Nucleic Acids Res. 48, D9–D16 (2020). 31602479 10.1093/nar/gkz899 PMC6943063

[87] M. Martin, Cutadapt removes adapter sequences from high-throughput sequencing reads. EMBnet J. 17, 10–12 (2011).

[88] A. Dobin, C. A. Davis, F. Schlesinger, J. Drenkow, C. Zaleski, S. Jha, P. Batut, M. Chaisson, T. R. Gingeras, STAR: Ultrafast universal RNA-seq aligner. Bioinformatics 29, 15–21 (2013). 23104886 10.1093/bioinformatics/bts635 PMC3530905

[89] N. L. Bray, H. Pimentel, P. Melsted, L. Pachter, Near-optimal probabilistic RNA-seq quantification. Nat. Biotechnol. 34, 525–527 (2016). 27043002 10.1038/nbt.3519

[90] M. S. Campbell, C. Holt, B. Moore, M. Yandell, Genome annotation and curation using MAKER and MAKER-P. Curr. Protoc. Bioinformatics 48, 4.11.1–4.11.39 (2014). 10.1002/0471250953.bi0411s48 PMC4286374 25501943

[91] M. Stanke, O. Keller, I. Gunduz, A. Hayes, S. Waack, B. Morgenstern, AUGUSTUS: Ab initio prediction of alternative transcripts. Nucleic Acids Res. 34, W435–W439 (2006). 16845043 10.1093/nar/gkl200 PMC1538822

[92] UniProt Consortium, UniProt: A worldwide hub of protein knowledge. Nucleic Acids Res. 47, D506–D515 (2019). 30395287 10.1093/nar/gky1049 PMC6323992

[93] I. Korf, Gene finding in novel genomes. BMC Bioinformatics 5, 59 (2004). 15144565 10.1186/1471-2105-5-59 PMC421630

[94] S. Götz, J. M. García-Gómez, J. Terol, T. D. Williams, S. H. Nagaraj, M. J. Nueda, M. Robles, M. Talón, J. Dopazo, A. Conesa, High-throughput functional annotation and data mining with the Blast2GO suite. Nucleic Acids Res. 36, 3420–3435 (2008). 18445632 10.1093/nar/gkn176 PMC2425479

[95] A. Conesa, S. Götz, J. M. García-Gómez, J. Terol, M. Talón, M. Robles, Blast2GO: A universal tool for annotation, visualization and analysis in functional genomics research. Bioinformatics 21, 3674–3676 (2005). 16081474 10.1093/bioinformatics/bti610

[96] D. Fontaneto, C. Q. Tang, U. Obertegger, F. Leasi, T. G. Barraclough, Different diversification rates between sexual and asexual organisms. Evol. Biol. 39, 262–270 (2012).

[97] E. A. Gladyshev, M. Meselson, I. R. Arkhipova, Massive horizontal gene transfer in bdelloid rotifers. Science 320, 1210–1213 (2008). 18511688 10.1126/science.1156407

[98] J.-F. Flot, B. Hespeels, X. Li, B. Noel, I. Arkhipova, E. G. J. Danchin, A. Hejnol, B. Henrissat, R. Koszul, J.-M. Aury, V. Barbe, R.-M. Barthélémy, J. Bast, G. A. Bazykin, O. Chabrol, A. Couloux, M. Da Rocha, C. Da Silva, E. Gladyshev, P. Gouret, O. Hallatschek, B. Hecox-Lea, K. Labadie, B. Lejeune, O. Piskurek, J. Poulain, F. Rodriguez, J. F. Ryan, O. A. Vakhrusheva, E. Wajnberg, B. Wirth, I. Yushenova, M. Kellis, A. S. Kondrashov, D. B. M. Welch, P. Pontarotti, J. Weissenbach, P. Wincker, O. Jaillon, K. Van Doninck, Genomic evidence for ameiotic evolution in the bdelloid rotifer Adineta vaga. Nature 500, 453–457 (2013). 23873043 10.1038/nature12326

[99] R. W. Nowell, P. Almeida, C. G. Wilson, T. P. Smith, D. Fontaneto, A. Crisp, G. Micklem, A. Tunnacliffe, C. Boschetti, T. G. Barraclough, Comparative genomics of bdelloid rotifers: Insights from desiccating and nondesiccating species. PLOS Biol. 16, e2004830 (2018). 29689044 10.1371/journal.pbio.2004830 PMC5916493

[100] E. G. J. Danchin, M.-N. Rosso, P. Vieira, J. de Almeida-Engler, P. M. Coutinho, B. Henrissat, P. Abad, Multiple lateral gene transfers and duplications have promoted plant parasitism ability in nematodes. Proc. Natl. Acad. Sci. U.S.A. 107, 17651–17656 (2010). 20876108 10.1073/pnas.1008486107 PMC2955110

[101] P. Abad, J. Gouzy, J.-M. Aury, P. Castagnone-Sereno, E. G. J. Danchin, E. Deleury, L. Perfus-Barbeoch, V. Anthouard, F. Artiguenave, V. C. Blok, M.-C. Caillaud, P. M. Coutinho, C. Dasilva, F. De Luca, F. Deau, M. Esquibet, T. Flutre, J. V. Goldstone, N. Hamamouch, T. Hewezi, O. Jaillon, C. Jubin, P. Leonetti, M. Magliano, T. R. Maier, G. V. Markov, P. McVeigh, G. Pesole, J. Poulain, M. Robinson-Rechavi, E. Sallet, B. Ségurens, D. Steinbach, T. Tytgat, E. Ugarte, C. van Ghelder, P. Veronico, T. J. Baum, M. Blaxter, T. Bleve-Zacheo, E. L. Davis, J. J. Ewbank, B. Favery, E. Grenier, B. Henrissat, J. T. Jones, V. Laudet, A. G. Maule, H. Quesneville, M.-N. Rosso, T. Schiex, G. Smant, J. Weissenbach, P. Wincker, Genome sequence of the metazoan plant-parasitic nematode Meloidogyne incognita. Nat. Biotechnol. 26, 909–915 (2008). 18660804 10.1038/nbt.1482

[102] A. Faddeeva-Vakhrusheva, K. Kraaijeveld, M. F. L. Derks, S. Y. Anvar, V. Agamennone, W. Suring, A. A. Kampfraath, J. Ellers, G. Le Ngoc, C. A. M. van Gestel, J. Mariën, S. Smit, N. M. van Straalen, D. Roelofs, Coping with living in the soil: The genome of the parthenogenetic springtail Folsomia candida. BMC Genomics 18, 493 (2017). 28659179 10.1186/s12864-017-3852-x PMC5490193

[103] G. Schönknecht, A. P. M. Weber, M. J. Lercher, Horizontal gene acquisitions by eukaryotes as drivers of adaptive evolution. Bioessays 36, 9–20 (2014). 24323918 10.1002/bies.201300095

[104] T. Guo, X.-W. Wang, K. Shan, W. Sun, L.-Y. Guo, The Loricrin-Like Protein (LLP) of phytophthora infestans is required for oospore formation and plant infection. Front. Plant Sci. 8, 142 (2017). 28232841 10.3389/fpls.2017.00142 PMC5298957

[105] Z. Yang, Y. Zhang, E. K. Wafula, L. A. Honaas, P. E. Ralph, S. Jones, C. R. Clarke, S. Liu, C. Su, H. Zhang, N. S. Altman, S. C. Schuster, M. P. Timko, J. I. Yoder, J. H. Westwood, C. W. dePamphilis, Horizontal gene transfer is more frequent with increased heterotrophy and contributes to parasite adaptation. Proc. Natl. Acad. Sci. U.S.A. 113, E7010–E7019 (2016). 27791104 10.1073/pnas.1608765113 PMC5111717

[106] J. R. Belyeu, M. Chowdhury, J. Brown, B. S. Pedersen, M. J. Cormier, A. R. Quinlan, R. M. Layer, Samplot: A platform for structural variant visual validation and automated filtering. *Genome Biol.* **22**, 161 (2021). 10.1186/s13059-021-02380-5 PMC8145817 34034781

[107] M. Mahmoud, N. Gobet, D. I. Cruz-Dávalos, N. Mounier, C. Dessimoz, F. J. Sedlazeck, Structural variant calling: The long and the short of it. Genome Biol. 20, 246 (2019). 31747936 10.1186/s13059-019-1828-7 PMC6868818

[108] J. Ruan, H. Li, Fast and accurate long-read assembly with wtdbg2. Nat. Methods 17, 155–158 (2020). 31819265 10.1038/s41592-019-0669-3 PMC7004874

[109] F. J. Sedlazeck, P. Rescheneder, M. Smolka, H. Fang, M. Nattestad, A. von Haeseler, M. C. Schatz, Accurate detection of complex structural variations using single-molecule sequencing. Nat. Methods 15, 461–468 (2018). 29713083 10.1038/s41592-018-0001-7 PMC5990442

[110] T. S. Korneliussen, A. Albrechtsen, R. Nielsen, ANGSD: Analysis of next generation sequencing data. BMC Bioinformatics 15, 356 (2014). 25420514 10.1186/s12859-014-0356-4 PMC4248462

[111] C. Trapnell, A. Roberts, L. Goff, G. Pertea, D. Kim, D. R. Kelley, H. Pimentel, S. L. Salzberg, J. L. Rinn, L. Pachter, Differential gene and transcript expression analysis of RNA-seq experiments with TopHat and Cufflinks. Nat. Protoc. 7, 562–578 (2012). 22383036 10.1038/nprot.2012.016 PMC3334321

[112] A. Löytynoja, N. Goldman, An algorithm for progressive multiple alignment of sequences with insertions. Proc. Natl. Acad. Sci. U.S.A. 102, 10557–10562 (2005). 16000407 10.1073/pnas.0409137102 PMC1180752

[113] G. Talavera, J. Castresana, Improvement of phylogenies after removing divergent and ambiguously aligned blocks from protein sequence alignments. Syst. Biol. 56, 564–577 (2007). 17654362 10.1080/10635150701472164

[114] A. A. Comeault, S. M. Flaxman, R. Riesch, E. Curran, V. Soria-Carrasco, Z. Gompert, T. E. Farkas, M. Muschick, T. L. Parchman, T. Schwander, J. Slate, P. Nosil, Selection on a genetic polymorphism counteracts ecological speciation in a stick insect. Curr. Biol. 25, 1975–1981 (2015). 26119745 10.1016/j.cub.2015.05.058

[115] R. Villoutreix, C. F. de Carvalho, V. Soria-Carrasco, D. Lindtke, M. De-la-Mora, M. Muschick, J. L. Feder, T. L. Parchman, Z. Gompert, P. Nosil, Large-scale mutation in the evolution of a gene complex for cryptic coloration. Science 369, 460–466 (2020). 32703880 10.1126/science.aaz4351

[116] M. Petersen, D. Armisén, R. A. Gibbs, L. Hering, A. Khila, G. Mayer, S. Richards, O. Niehuis, B. Misof, Diversity and evolution of the transposable element repertoire in arthropods with particular reference to insects. BMC Evol. Biol. 19, 11 (2019). 30626321 10.1186/s12862-018-1324-9 PMC6327564

