# Supplementary material for: Convergent consequences of parthenogenesis on stick insect genomes
Source: Sci Adv. 2022 Feb 23;8(8):eabg3842. doi: 10.1126/sciadv.abg3842 (PMC8865771; doi:10.1126/sciadv.abg3842)
Supplement: Supplementary file 1 — Supplementary Text Figs. S1 to S14 Tables S1 to S9 References [file sciadv.abg3842_sm.pdf]

Science Advances
AAAS

Supplementary Materials for

Convergent consequences of parthenogenesis on stick insect genomes

Kamil S. Jaron*, Darren J. Parker*, Yoann Anselmetti, Patrick Tran Van, Jens Bast, Zoé Dumas, Emeric Figuet, Clémentine M. François, Keith Hayward, Victor Rossier, Paul Simion, Marc Robinson-Rechavi, Nicolas Galtier, Tanja Schwander*

*Corresponding author. Email: darrenjames.parker@unil.ch (D.J.P.); kamil.jaron@ed.ac.uk (K.S.J.); tanja.schwander@unil.ch (T.S.)

Published 23 February 2022, *Sci. Adv.* **8**, eabg3842 (2022)
DOI: 10.1126/sciadv.abg3842

The PDF file includes:

Supplementary Text
Figs. S1 to S14
Tables S1 to S9
References

Other Supplementary Material for this manuscript includes the following:

Data S1

Supplementary Text

Assembly and annotation pipelines

Paired-end raw reads were trimmed according to sequencing quality and matched to known Illumina sequencing adapters using Trimmomatic (v.0.36) (*81*). Leading and trailing bases below quality 9 were removed. Reads were scanned using a 4-base sliding window, trimmed when the average quality dropped below 15, and discarded if read length dropped below 96bp (Parameters: PE ILLUMINACLIP: all-adapters.fa:3:25:6 LEADING:9 TRAILING:9 SLIDINGWINDOW:4:15 MINLEN:96). The raw mate-pair reads were de-linked and reverse complemented using NxTrim (v. 0.4.1) (*82*) with the parameter "--preserve-mp". Unlinked pairs without identified adapter sequence, called unknown pairs, were also considered as valid mate pairs as they had a similar distribution of insert sizes as mate pairs with identified linker sequence.

Filtered paired-end reads were *de novo* assembled using ABySS (v. 1.9.0) (*47*, *83*) with default parameters and k-mer sizes predicted to be optimal using kmergenie (*84*). The k-mer sizes were 83, 87, 83, 87, 83, 89, 81, 81, 65 and 87 for *Timema poppensis*, *T. douglasi*, *T. californicum*, *T. shepardi*, *T. cristinae*, *T. monikensis*, *T. barmani, T. tahoe, T. podura and T. genevievae* respectively. Assembled contigs longer than 250 bases were scaffolded using BESST (v. 2.2.5) (*48*) with default parameters and gap-filled with GapCloser (v. 1.12-r6), a module of the SOAP package (*85*).

Genome assemblies were decontaminated using BlobTools (v. 0.9.19.5) (*49*). Hit files were generated after a BlastN (v. 2.6.0) (*69*) against the NBCI nt database (v 2016-06) (*86*), searching for hits with sequence identity above 85% and an e-value below 1e-25 (Parameters: -task megablast -culling_limit 5 -evalue 1e-25 -perc_identity 85). Scaffolds without hits to metazoans were removed from the assemblies. The genome assembly completeness was assessed with BUSCO (v. 3.0.2) (*21*) against the insecta_odb9 lineage and the -long option. For genome annotation, we took a total of 231 publically available RNA-seq libraries (Bioproject Accessions: PRJNA679785, PRJNA678950, PRJNA380865, PRJNA392384) for *Timema* from different tissues, sexes and developmental stages as expression evidence (min per species = 12, see Table S8) (*34*, *50*, *51*). Before mapping reads to the genomes, adapter sequences

were trimmed from raw reads with CutAdapt (v. 1.15) (*87*). Reads were then quality trimmed using Trimmomatic (v. 0.36) (*81*), clipping leading or trailing bases with a phred score of <10 from the read, before using a sliding window from the 5' end to clip the read if 4 consecutive bases had an average phred score of <20. Any reads with a sequence length of <80 after trimming were discarded. All trimmed RNA-seq reads were then mapped against the genomes as single end reads using STAR (v. 2.5.3a) (*88*) under the "2-pass mapping" mode and default parameters. The STAR outputs were then used to produce transcriptome assemblies using Trinity (v. 2.4.0) (*52*) "genome guided" mode (Parameters: --genome_guided_max_intron 100000 --SS_lib_type R). Finally, the transcriptome assemblies were filtered following Trinity developers recommendations (https://github.com/trinityrnaseq/trinityrnaseq/wiki/Trinity-FAQ): Briefly, filtered RNA-seq reads were mapped back against the transcriptomes using Kallisto (v. 0.43.0) (*89*) with options --bias and --single, then transcripts with at least 1 TPM in any sample were retained.

Genome scaffolds >1000 bp were annotated, protein coding genes were predicted using MAKER (v. 2.31.8) (*53*) in a 2-step iterative way as described in Campbell *et al.* (*90*) with minor modifications following author recommendations. For the first iteration, genes were predicted using Augustus (v. 3.2.3) (*91*) trained with the BUSCO results. A combination of UniProtKB/Swiss-Prot (release 2018_01) (*92*) and the BUSCO insecta_odb9 proteome were used as protein evidence. The Trinity assembled RNA-seq reads (described above) were used as transcript evidence. The resulting gene models were then used to retrain Augustus as well as SNAP (v. 2013.11.29) (*93*) and a second iteration was performed. Predicted protein coding genes were then functionally annotated using Blast2GO v5.5.1 (*94*, *95*) with default parameters against both the NCBI non-redundant arthropods protein database, and the *Drosophila melanogaster* (drosoph) database, to produce two sets of functional annotations, one derived from all arthropods and one specifically from *Drosophila melanogaster*.

Horizontal Gene Transfers (HGTs) are not facilitated by parthenogenesis

Genomic analyses of bdelloid rotifers, a group that likely persisted and diversified in the absence of canonical sex for over 40 million years (*96*), revealed that bdelloids carry an unusually large amount (6.2% - 9.1%) of horizontally acquired genes compared to sexual lophotrochozoans (0.08% - 0.7%) (*16*, *97–99*). Unusually high proportions of HGT-derived genes were also identified in parthenogenetic root-knot nematodes (*100*, *101*) and springtails (*102*). These findings led to the suggestion that parthenogenesis might favor the retention of horizontally acquired genes, and may perhaps confer adaptive benefits that could compensate for the absence of recombination and outcrossing (*100*), although such patterns are not shared by most other parthenogenetic animal genomes (*16*). Analyzing HGT events in *Timema* provided no evidence for parthenogenesis facilitating the retention of HGTs. We identified 55 putative HGT events in the 10 *Timema* species, with up to 50 sequences each, for a total of 704 HGT-derived sequences (351 in the five sexual species vs. 353 in the five parthenogenetic species). The genome of each *Timema* species included approximately 70 HGT-derived sequences, comparable to values from metazoa in general (*103*). All putative HGT families were shared by at least six *Timema* species, and only one putative HGT event occurred in a specific clade (HGT family shared between two sexual and two parthenogenetic species of the Northern clade) while all other HGT events were shared between at least two clades of *Timema* (Fig. S7).

Of note, out of the 55 HGT families, 34 featured significant similarities with sequences from two plant pathogens (*Phytophthora infestans* and *Pythium ultimum*), and displayed a high-glycine content, due to many 'GGG' repeats. This repeated motif is very similar to the loricrin-like protein described in *Phytophthora infestans* by Guo et al. (*104*), which is suggested to be involved in plant infection.

Out of the 21 remaining families, only 4 showed a phylogenetic pattern consistent with an old HGT event, sometimes shared with *Zootermopsis nevadensis* (the closest species in our reference database). However, the terminal branches leading to the putatively-transferred sequences were too long for a reliable identification of the donor species. The phylogenetic evaluation of the other families was not conclusive, as commonly observed in HGT detection studies (e.g. see (*105*)).

The pipeline we used for HGT detection only retains HGT candidates if they are on a scaffold with a CDS with close homology to an arthropod CDS (see Methods). While this approach avoids misidentifying scaffolds entirely derived from non-metazoans as HGT candidates, mis-assembled chimeric scaffolds would be retained. To assess whether our HGT candidates were such false-positives, we used a PacBio long-read dataset (~32x coverage) for one of the parthenogenetic species (*T. douglasi*; PRJNA673001). We mapped the PacBio reads to our *T. douglasi* reference genome using ngmlr (v0.2.7) with default parameters. To verify physical linkage we examined the eight *T. douglasi* scaffolds which contained HGT candidates flanked by regions containing ‘confident-arthropod’ assigned genes. Of these eight candidates, there was clear linkage (i.e. the full scaffold was covered by overlapping long reads, Fig. S8A, S8B) for six of them. One scaffold showed a clear sign of chimerism (i.e. a region with no overlapping long reads, Fig. S8C), while for the remaining one, chimerism is likely but unclear (only supported by a single overlapping long read, Fig. S8D). Overall, these analyses show that the majority of HGT candidates in *Timema* are not derived from chimeric scaffolds.

Analysis of heterozygosity for SNPs and SVs

We present two estimates of heterozygosity, one based on a reference-free technique (kmer spectra analysis using Genomescope (v. 2) (*22*), the other using sequencing reads mapped to reference genomes to call SNPs with GATK ((*62*), see Methods).

The kmer spectra of all sexual species displayed distinct haploid coverage peaks representing heterozygous kmers (Fig. S9A), contrasting with the kmer spectra of parthenogenetic species, where no distinct peaks were visible (Fig. S9B). To confirm that no heterozygous kmers were present at the expected haploid coverage in parthenogens, we used Smudgeplot (*22*), a technique to extract closely related kmer pairs representing heterozygous and paralogous kmer pairs. While in the sexual species, kmers from the 1n peak paired together in heterozygous kmer pairs (AB smudge on Fig. S9C), no diploid kmer pairs were detected in the parthenogenetic species (Fig. S9D). We conclude that heterozygosity estimates for the parthenogenetic species cannot be based on k-mer spectra analyses because the heterozygosity levels are too low to reliably fit the distribution estimating haploid kmers in the kmer spectra. Unreliable heterozygosity estimates based on k-mer spectra

analyses for species with very low heterozygosity was already reported in Jaron et al. (*16*), suggesting that with the current quality of sequencing data, kmer methods do not have resolution for very small heterozygosity levels.

Because we could not estimate heterozygosity of parthenogens using kmer-spectra analyses, we estimated nucleotide heterozygosity using SNP calling. It is important to note, however, that this method generates an underestimation of heterozygosity given our fragmented reference genomes (Table S3) and relatively modest coverage (~14 - 21x) of re-sequenced samples. Therefore, our SNP heterozygosity estimates in *Timema* are useful for comparing sexual and parthenogenetic species, but are not accurate estimates of heterozygosity in *Timema* (which range from 0.36 to 2.16% for sexual species, i.e., 2-6 times higher than the SNP-based estimates, Fig. 2). In agreement with genome profiling, we find very low, nearly negligible levels of heterozygosity in parthenogenetic species (Fig. 2; % of heterozygous bases: 0.00055% for *T. tahoe*, 0.00156% for *T. shepardi*, 0.00057% for *T. monikensis*, 0.00112% for *T. douglasi* and 0.00033% for *T. genevievae*). Furthermore, a large portion of the heterozygous SNP calls in parthenogens showed an unexpectedly high coverage (Fig. S6). This excess coverage of heterozygous positions in parthenogens suggests that heterozygous sites in parthenogens largely stem from merged paralogs, further supporting that a very large proportion (or maybe even all) of the called heterozygous variants in parthenogens are just artifacts of the SNP calling pipeline using whole genome data.

We further investigated if there were any heterozygous structural variations in parthenogenetic *Timema*, as those could be potentially hidden to SNP analysis. Consistent with the previous two analyses, the SV heterozygosity levels were substantially lower in the parthenogens than in their sexual sister species (Fig. 2). However, we also detected a non-negligible amount of heterozygous structural variants. We therefore manually curated all heterozygous structural variants found in *T. monikensis* using samplot (v1.0.1) (*106*), but did not find a single variant clearly supported by reads (results not shown). Since structural variant calling from short read data has a high rate of false positives regardless of the method used (*107*), we decided to verify variants using a PacBio long-read dataset (~32x coverage) for one of the parthenogenetic species (*T. douglasi;* PRJNA673001). We assembled the long-read

data of *T. douglasi* using Redbean (formerly wtdbg; v2.5) assembler (*108*) with parameters recommended for moderately sized genomes: -L 1000 -x preset3 -g 1300m. This genome assembly was used for SV calling using ngmlr (v0.2.7) and the Sniffles (v1.0.11) pipeline (*109*) with default parameters for SV calling using long read data. In total, we found only 6 heterozygous SVs: 4 deletions and 2 insertions. We visualized the SVs alongside their read support using samplot and found that none were well supported (Fig. S10) suggesting that the heterozygous SVs called using short read data represent noise in the absence of a signal from real heterozygous SVs.

In conclusion, we used four complementary approaches based on three different data sources: kmer spectra analysis on raw sequencing reads of the reference individuals, SNP and SV heterozygosity estimates using variant calling based on resequencing data, and finally a long read dataset of *T. douglasi*, which was independently assembled and is therefore free of any potential biases introduced in a short read assembly. Our analyses comprehensively show the absence of heterozygous loci in the parthenogenetic *Timema* genome assemblies. Residual heterozygosity could be potentially found in repetitive regions, such as centromeres and telomeres (see also above), as all our effort to detect heterozygosity focused on alleles with 1n coverage (half of the genome coverage). However, detecting heterozygosity in such regions requires chromosome-scale assemblies based on long-read sequencing technologies, which are currently not available for parthenogenetic *Timema*.

Locating microsatellite markers in the genome assemblies

Previous research, based on microsatellite markers, suggested that oogenesis in parthenogenetic *Timema* was functionally mitotic, as there was no loss of heterozygosity between females and their offspring (*17*). Yet our genome data reveal complete or almost complete homozygosity in the genome assemblies of parthenogens (see main text). The most likely reconciliation of these contrasting results is that heterozygosity is maintained in only a small portion of the genome, for example the centromeres or telomeres, or between paralogs.

To investigate these possibilities, we searched for the primer pairs used to amplify the nine microsatellites in the genome assembly v1.3 of the sexual species *T. cristinae*

from Nosil et al (*32*). This assembly is currently the most complete and least fragmented *Timema* assembly available, and the microsatellites used by (*17*) were originally developed for *T. cristinae*. We used Blast to find primer pairs <500 bp apart, on opposite strands, and retained significant hits with at least 80% of the primer sequences covered. We then verified whether the retained hits comprised the expected microsatellite repeat motif.

Using this approach, we were able to locate six of the nine microsatellites in the v1.3 assembly (Table S9). Two of the six microsatellites had multiple hits in the genome (Table S9). In combination, these results support the idea that microsatellite heterozygosity detected in *Timema* parthenogens may be a combination of heterozygosity in centromere or telomere regions (microsatellites not detected in the assembly) and heterozygosity between paralogs (microsatellites with multiple copies in the *T. cristinae* assembly).

Polymorphism in parthenogenetic and sexual *Timema* populations

To compare the distribution of polymorphism along different genomes, we mapped population-level variation for SNPs and SVs inferred from 2 to 5 re-sequenced individuals per population to our species-specific reference genomes (see main text). We then anchored our reference genome scaffolds to the 12 autosomal linkage groups of a previously published assembly of the sexual species *T. cristinae* (v1.3 from Nosil et al. (*32*)) using MUMmer (v. 4.0.0beta2) (*65*) (see Methods for details). Note that we excluded LG13, classified as the X chromosome in Nosil et al (*32*), because coverage comparisons using publicly available genomic data from males (bioproject accession: PRJNA725673) and females (this study) revealed that LG13 did not correspond to the X. We also removed X-linked scaffolds assigned to autosomal LGs in the v1.3 *T. cristinae* assembly and used this “cleaned” set of linkage groups (referred to as v1.4) in all our analyses with positional information. Depending on the species, we were able to anchor between 59 and 558 Mbp of our genomes to the *T. cristinae* LGs.

We also examined how genetic variation was distributed between individuals by producing phylogenetic trees for the re-sequenced and reference individuals of each sexual-parthenogenetic sister species pair. Sequences for re-sequenced individuals were obtained by mapping reads of each re-sequenced individual to the reference

genomes with BWA-MEM (v0.7.15) (*71*). Multi-mapping and poor quality alignments were filtered (removing reads with XA:Z or SA:Z tags or a mapq < 30). We removed PCR duplicates with Picard (v. 2.9.0) (http://broadinstitute.github.io/picard/) and performed indel realignment with GATK (v. 3.7) (*62*). Genomic sequences for each re-sequenced individual were then generated using AngsD (v. 0.921) (-doCounts 1 -doFasta 2) (*110*) with a minimum depth of 5 and a maximum depth of twice the median genome coverage. Coding sequences of 1-to-1 orthologs (2198) were extracted using gffread from the Cufflinks (v. 2.2.1) package (*111*). These sequences were codon-aligned using PRANK (v.100802) (*112*) concatenated together, and filtered with GBlocks (v. 0.91b, type = codons, minimum block length = 12) to remove large alignment gaps and blocks of Ns (*113*). Trees were generated with RAxML (*61*), with a GTR+gamma model with 40 rate categories for each codon position (i.e. each codon position (1st ,2nd, 3rd) was partitioned to allow a distinct model to be fitted to it) to produce an ML tree with 1000 bootstraps.

Polymorphism and color morphs on LG8 in the species *T. californicum* and *T. monikensis*

We found very high population polymorphism for both SNPs and SVs on LG8 in *T. californicum* and *T. monikensis* (Fig. 3B). A site frequency spectrum (SFS) in *T. californicum* based on the SNPs called in the 5 resequenced individuals revealed that the polymorphism in this species was likely generated by the presence of two distinct haplotypes. The genotype structure for the 5 individuals was similar for a large portion of SNPs on LG8 (1/1 0/0 0/1 0/1 0/1; Fig. S11A), with three individuals heterozygous, and the two remaining ones homozygous for alternative alleles (Fig. S11A). The LG8 SNP genotypes further matched the grey versus green color morphs, with the grey morph known to have recessive inheritance (*114*): the individual used to build the reference genome (SNP genotypes 0/0) was grey, as was resequenced individual 2 (Tcm_02) with 0/0 genotypes. The four remaining resequenced individuals (with 1/1 or 0/1 genotypes) were green. The size of the putative haplotypes associated with green or grey morphs is considerable, spanning approximately 24 Mbp on LG8. The genotypes at LG8 in *T. monikensis* were also correlated with color morphs. Brown or beige melanistic morphs featured 0/0 genotypes in the high polymorphism region of LG8, while green individuals had 1/1 genotypes (Fig. S11B).

We also investigated the presence of an approximately 0.5 Mb deletion on LG8, suggested by Villoutreix et al. (*115*) to determine the green morphs in *T. californicum,* in our ~24 Mbp long haplotypes. Because our reference genome was based on a grey individual (which would be homozygous for the deletion-free haplotype), we expected to observe normal coverage for this region in the re-sequenced grey individual, zero coverage in the green re-sequenced individual homozygous for the alternative haplotype, and half the coverage in the three heterozygous individuals. We observed a coverage reduction in all individuals at the focal region, which could be due to an enrichment in repetitive sequences (non-uniquely mapping reads are not included for coverage estimations). Nevertheless, the grey individual featured somewhat higher coverage, consistent with the deletion suggested by Villoutreix et al. (*115*) (Fig. S12). Further studies are required to characterize the contribution of the two divergent, ~24 Mbp long haplotypes, and the putative 0.5 Mbp deletion in one of the haplotypes, to color polymorphism in *T. californicum*.

Transposable element activity in the *Timema* genus

The overall TE content was very similar in all ten species (20 - 23.6%), and different only by 1.3% between the two clades separating at the ancestral node (see main text). This can be explained by largely inactive TEs, or well balanced transposition and excision rates that keep the overall TE contents relatively stable. Thus, evidence of continuous and recent TE activity would support the second explanation, whereas absence thereof would be better consistent with the first explanation. To detect recent and past TE activity in *Timema*, we analysed TE sequence divergence landscapes in each species using Repeatmasker utility scripts (see Methods).

We found that TE copies with high similarity (indicating recent duplication) had low overall abundance, supporting the idea that recent TE activity is low across *Timema* species (Fig. S13). Between sexual and parthenogenetic sister species, highly similar TE copies appear to be more abundant in parthenogenetic than sexual species. This result however is likely artefactual, caused by the systematically better genome assembly quality in the parthenogenetic than sexual species (Table S3), meaning similar TE copies will be collapsed more often in the sexual than parthenogenetic genome assemblies. This effect can be shown by the fact that the coverage of low

divergence TEs is systematically higher for sexual species than parthenogenetic species, but this is not seen for bins with higher divergence (Fig. S14).

Overall our analyses suggest that the consistency of TE content across *Timema* is due to low TE activity in the genus. Such low activity also means that even if parthenogenesis affected TE accumulation, parthenogenesis would have to be much older than in *Timema* species to generate detectable effects on TE contents. This result may be applicable more generally if parthenogenetic taxa are typically present in groups that have stable TE content and/or low TE activity. Currently, few studies have examined TE content for multiple species in a genera (but see (*116*)) meaning future work is needed to determine if TE content is typically stable at the genus level (as we find in *Timema*), or if parthenogenetic taxa are more likely to be present in groups with stable TE content (*16*).

Finally, while the overall similarity of TE content in *Timema* is most consistent with low amounts of recent TE activity, we cannot formally exclude alternative explanations such as well balanced transposition and excision rates without direct studies of TE activity in *Timema*.

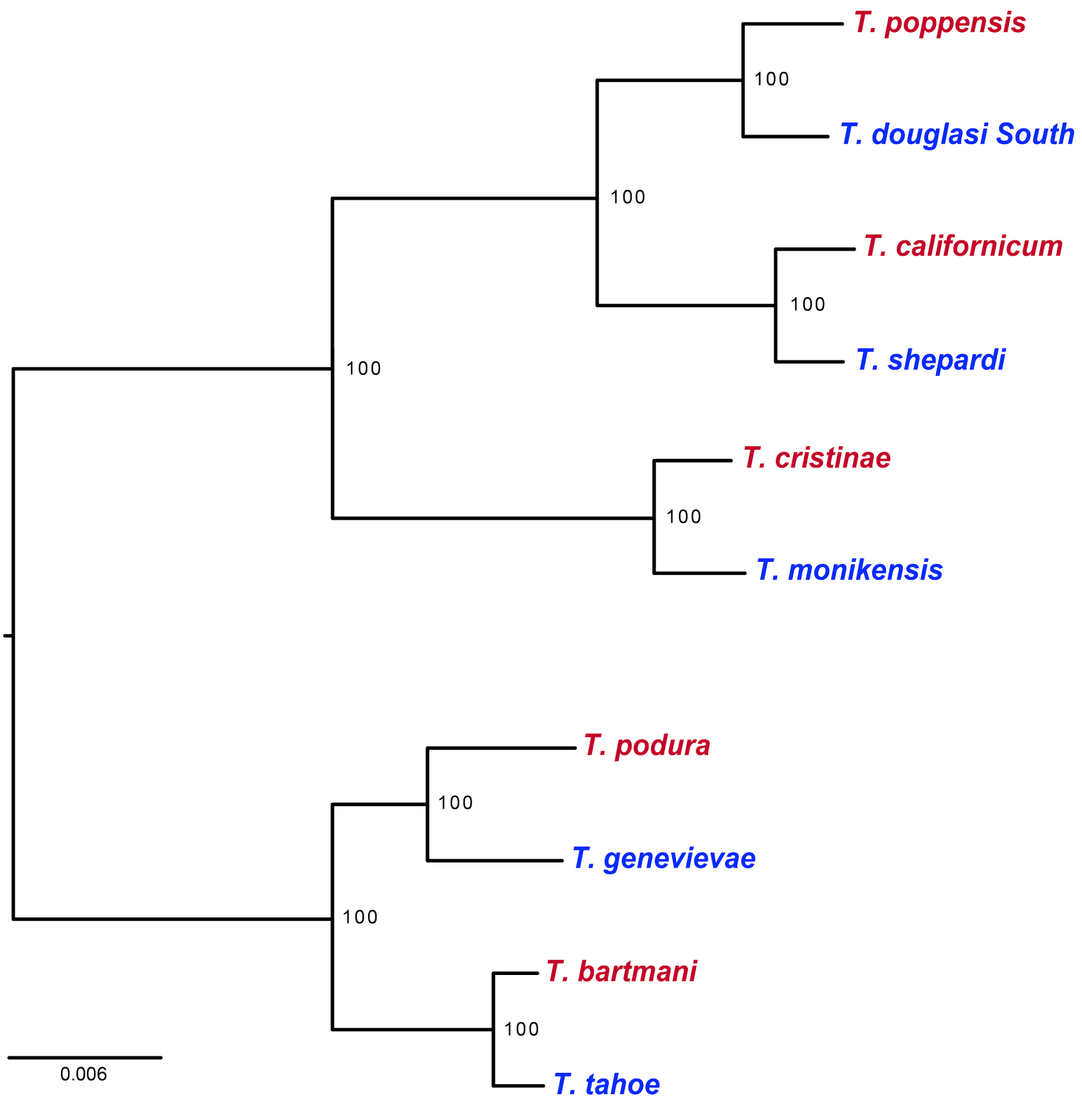

Fig. S1. Timema phylogeny. Maximum likelihood tree based on 2377398 orthologous coding DNA positions (from 3975 orthologs), rooted at the midpoint. Branch lengths represent the mean number of substitutions per site. Node labels indicate branch support (%) from 1000 bootstrap replicates. Othologs were aligned using MCoffee (v11.00.8cbe486) (*74*) which was run with the following aligners: mafft_msa, muscle_msa, clustalo_msa (*75*), and t_coffee_msa (*76*). Alignments were concatenated together, and filtered with GBlocks (v. 0.91b, type = codons, minimum block length = 12) to remove large alignment gaps and blocks of Ns (*113*). The tree was then generated with RAxML (*61*), with a GTR+gamma model with 40 rate categories for each codon position.

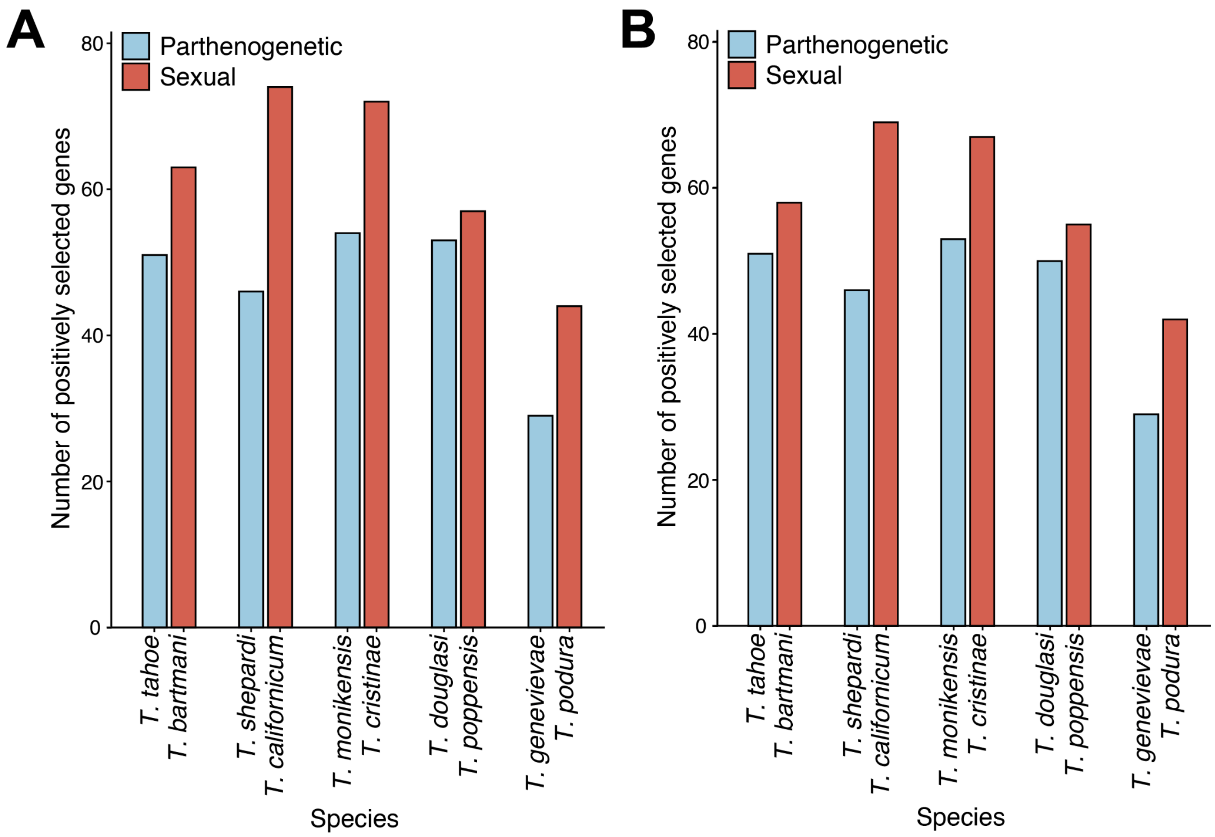

Fig. S2. Number of genes showing evidence for positive selection with different thresholds. A. Number of genes showing evidence for branch-site positive selection on terminal branches with a q-value threshold of 0.01 (binomial GLMM p-value for reproductive mode = 0.001). **B.** Number of genes showing evidence for branch-site positive selection on terminal branches with a q-value threshold of 0.01 when genes with polymorphic, positively-selected sites were excluded (binomial GLMM p-value for reproductive mode = 0.003).

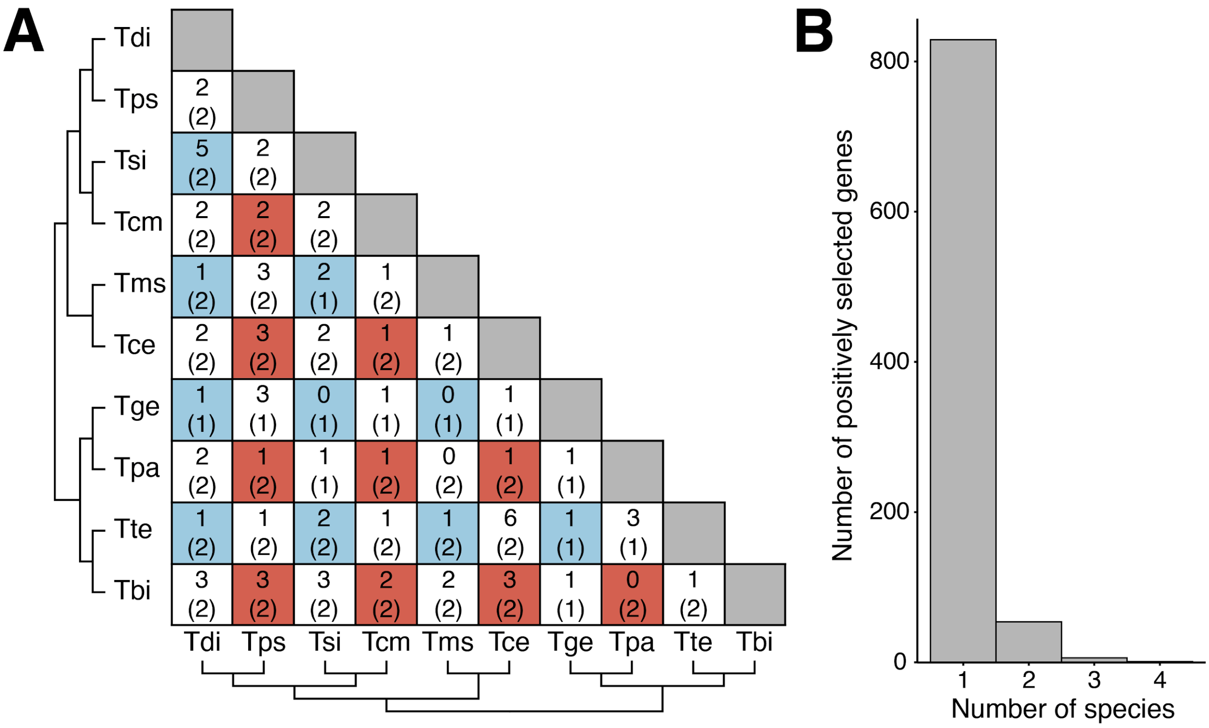

Fig. S3. Positively selected genes are shared between few species. A. Matrix showing pairwise overlap of positively selected genes with the number of genes expected by chance given in parentheses. Red cells indicate the overlap between two sexual species, blue between two parthenogenetic species, and white between one sexual and one parthenogenetic species. **B.** Number of species positively selected genes are found in.

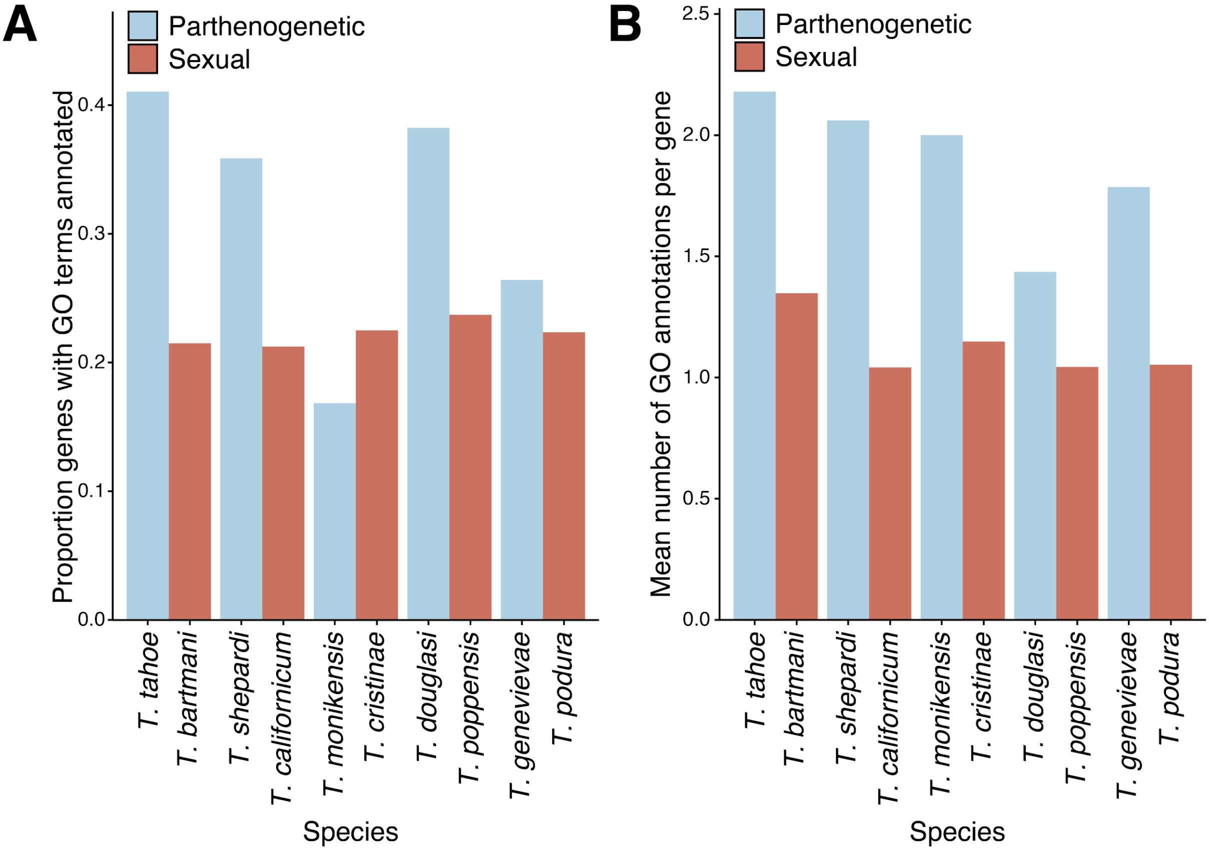

Fig. S4. Positively selected genes in sexual species have fewer annotations than in parthenogenetic species. A. Proportion of positively selected genes with at least 1 GO term (biological processes) annotated. **B.** Mean number of GO terms annotated in positively selected genes with at least 1 GO term (biological processes) annotated.

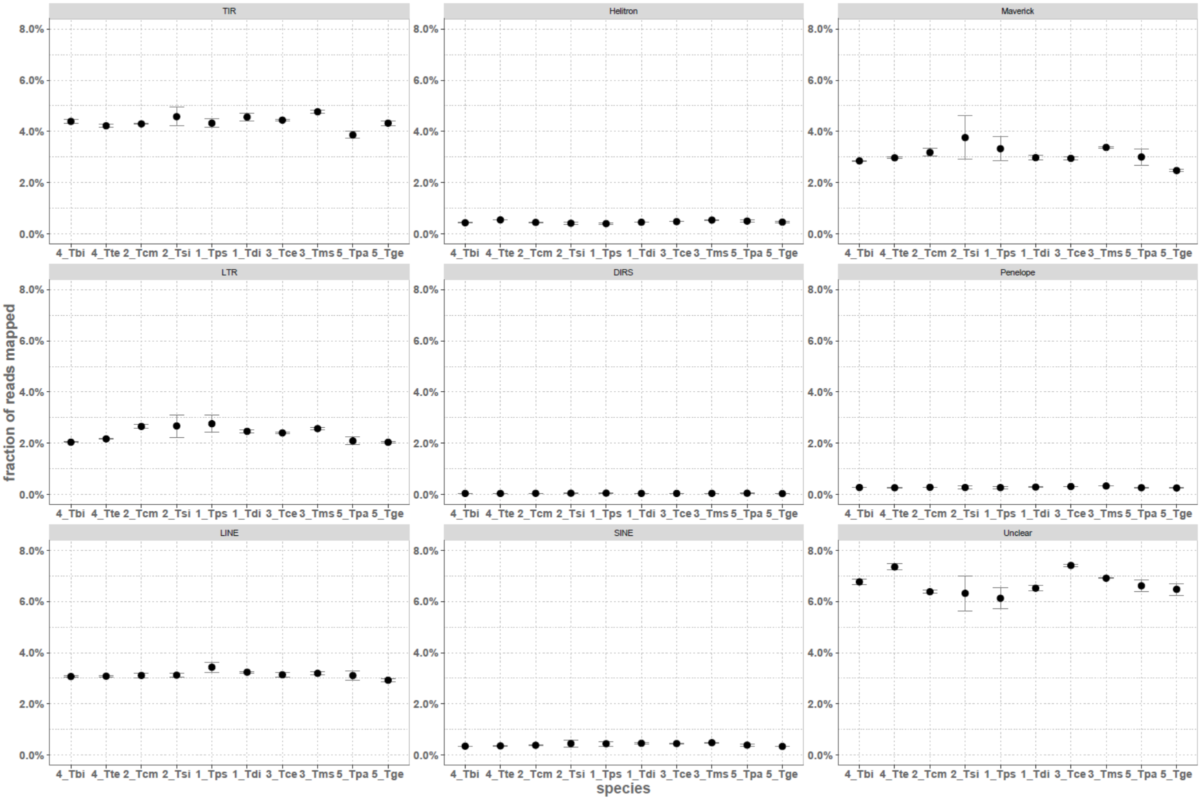

Fig. S5. Genomic transposable element abundance, separated by TE orders. Error bars represent standard deviation across the six (three for Tsi) sequenced genomes in each species. Species are abbreviated as follows: Tbi = *T. bartmani*, Tce = *T. cristinae*, Tps = *T. poppensis*, Tcm = *T. californicum*, Tpa = *T. podura*, Tte = *T. tahoe*, Tms = *T. monikensis*, Tdi = *T. douglasi*, Tsi = *T. shepardi*, and Tge = *T. genevievae.*

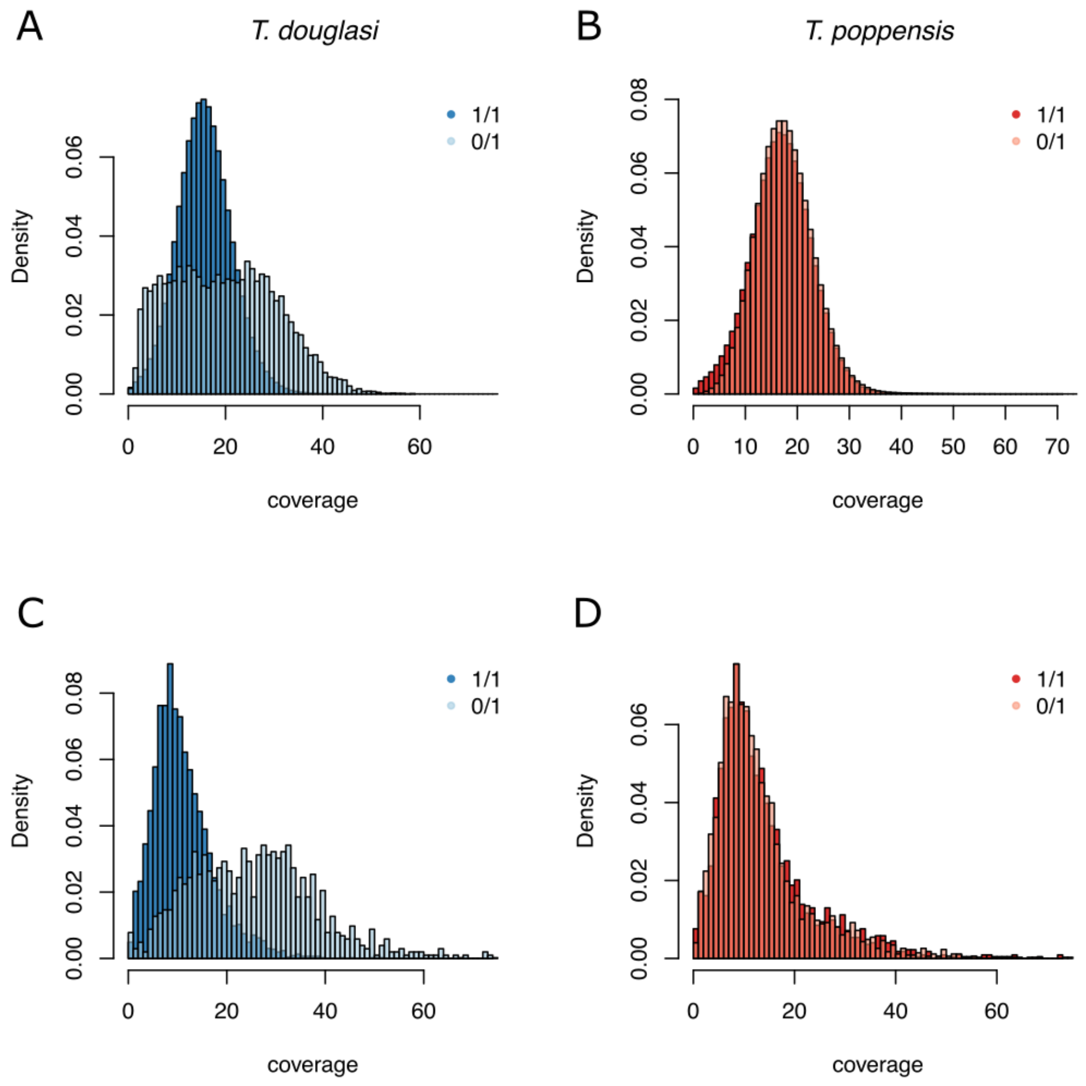

Fig. S6. Coverage of SNPs in *T. douglasi* and *T. poppensis*. Densities of coverages supporting **A.** SNPs found in the homozygous state (1/1), or heterozygous (0/1) in *T. douglasi*. **B.** In sexual *T. poppensis.* **C.** Densities of split read coverage support of SVs in homozygous or heterozygous states in *T. douglasi* and **D.** *T. poppensis*. Both heterozygous SNPs and heterozygous SVs show unexpected coverage distributions in parthenogenetic *T. douglasi* (blue), while coverages supporting SNPs in sexual *T. poppensis* (red) are independent of the genotype. There is a small difference in homozygous and heterozygous SV coverages in sexuals, suggesting that at least some fraction of those heterozygous SVs are also false positives. However, overlap of the two distributions is much greater than in the case of parthenogenetic *T. douglasi* (panel C).

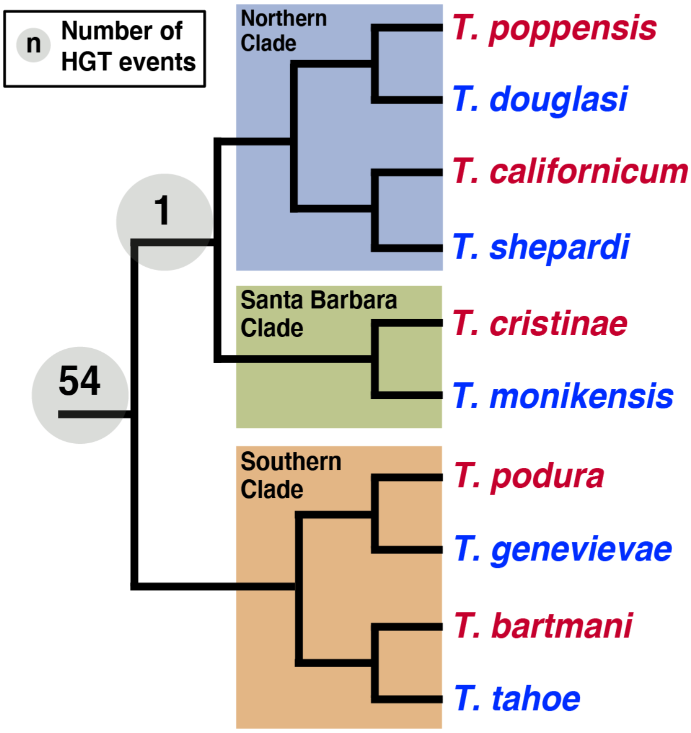

Fig. S7. HGT events placed on the *Timema* phylogeny. All HGT candidates were detected in more than one species, most of them (54 out of 55) were shared by species present in each of the three different *Timema* clades and therefore were probably acquired prior to the diversification of the genus. Only a single HGT candidate was not detected in the Southern clade, which could indicate a later acquisition or a loss in the common ancestor of the Southern clade.

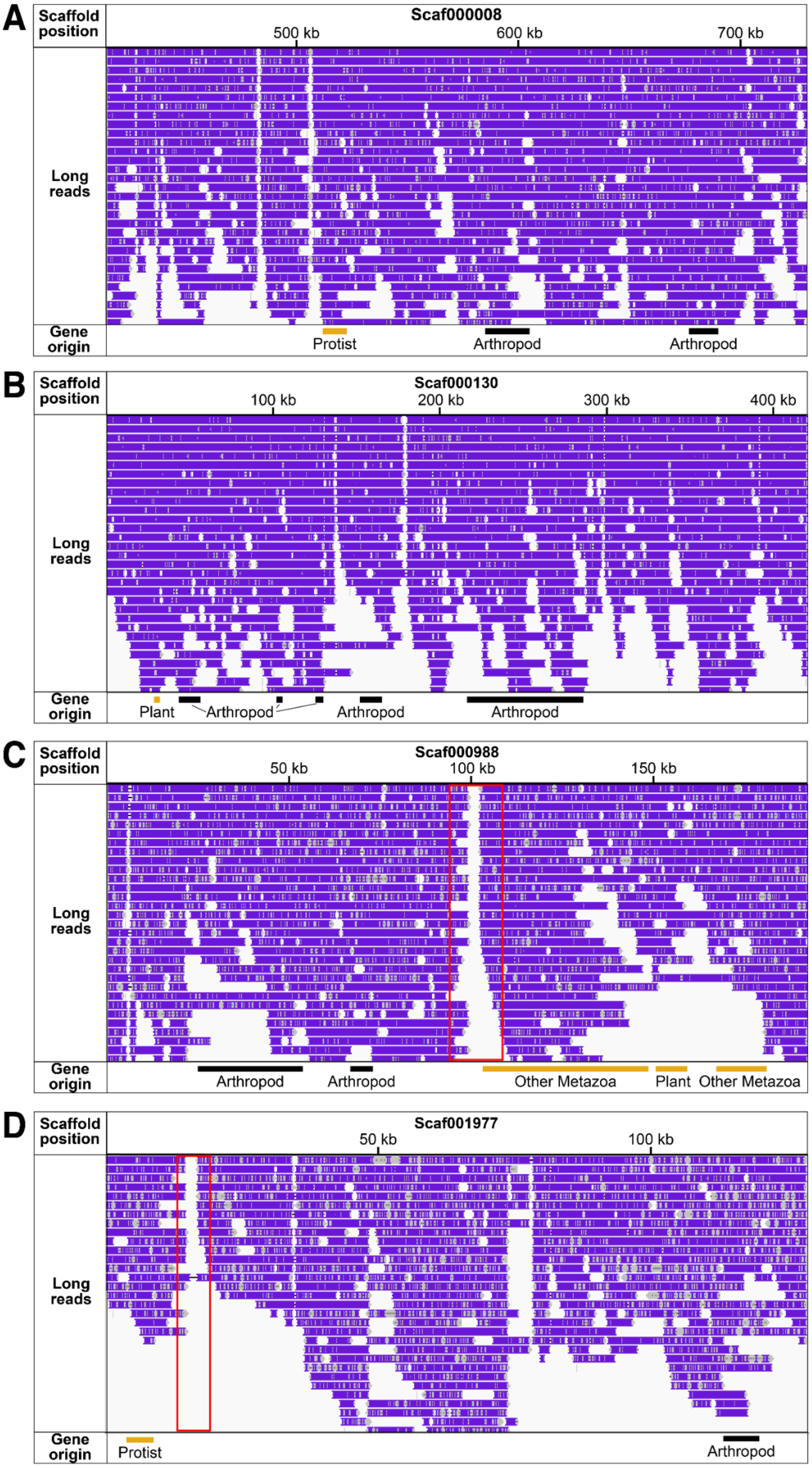

Fig. S8. Long read support of linkage between HGT candidates and confident arthropod genes. Examples of well supported linkage are shown in panels **A** and **B**.

A likely chimeric scaffold is shown in panel **C**, marked by the red box. A potentially chimeric scaffold with only a single long read supporting the linkage is shown on panel **D**.

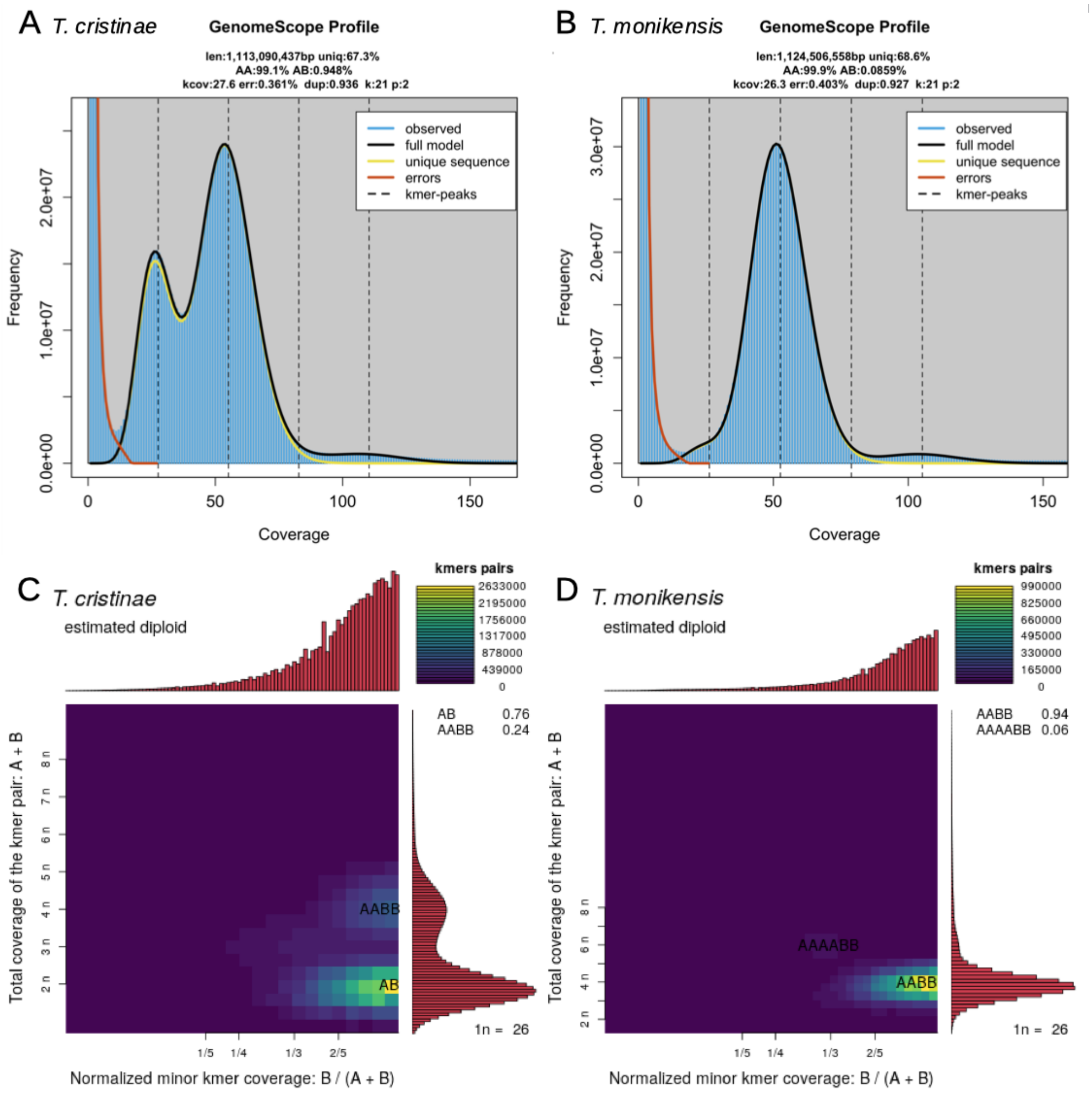

Fig. S9. Genome profiling examples for a sexual (*T. cristinae*, panels A and C) and a parthenogenetic (*T. monikensis*, panels B and D) *Timema* species.

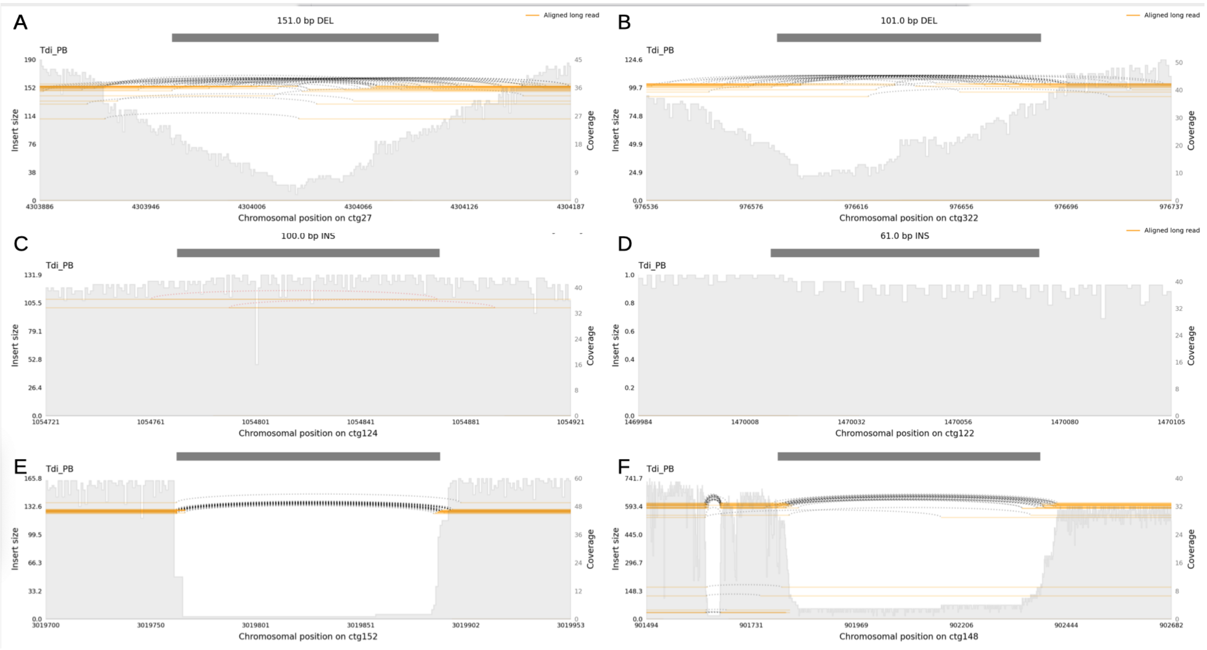

Fig. S10. All 6 heterozygous SVs called in the *T. douglasi* long read dataset. SVs on panels **A** and **B** are located in repetitive regions which is causing the uneven distribution of coverages and variable lengths of gaps. Variants on panels **C - F** are not supported by approximately half of the reads. Variant **C** is probably due to rare chimeric reads, and variant **D** does not seem to have any support at all. Conversely, SVs on panels **E** and **F** have very low support for the reference sequence. See examples provided in the manual of samplot for comparison to a well supported heterozygous SV.

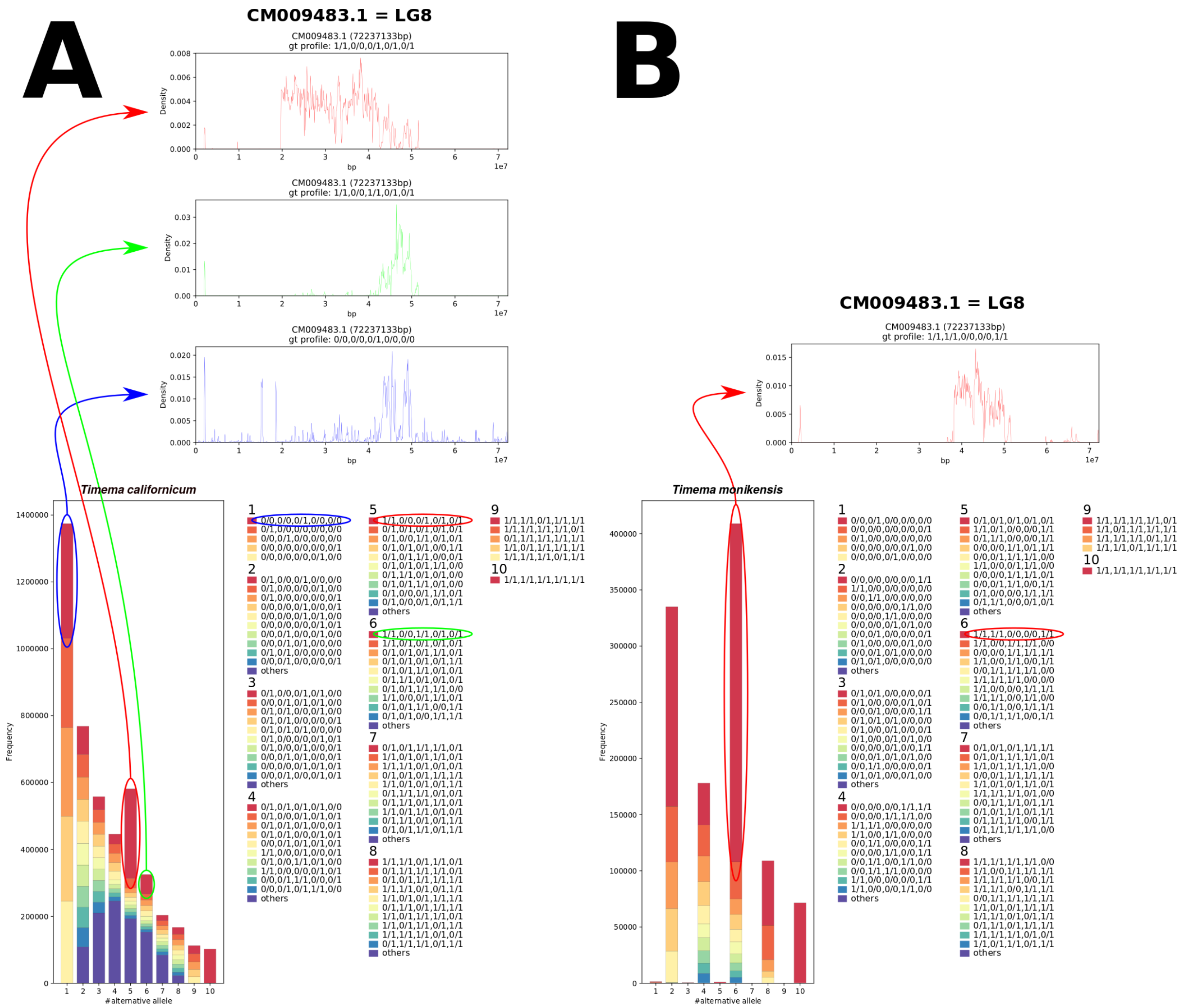

Fig. S11. Site Frequency Spectrum for the five re-sequenced individuals of *T. californicum* (A) and *Timema monikensis* (B). Site Frequency Spectra were generated with Pop-Con (https://github.com/YoannAnselmetti/Pop-Con), indicating the genotype distributions for each count of alternative alleles. For *T. californicum*, the peak at count 5 is generated by the overrepresented genotype structure 1/1 0/0 0/1 0/1 0/1, and almost all SNPs with this structure (97.34%) map to LG8, suggesting the presence of two divergent haplotypes on LG8. For *T. monikensis*, we observed a similar overrepresentation at allele count 6, for the genotype structure 1/1 1/1 0/0 0/0 1/1, and most of the SNPs with this structure (78.43%) map to LG8 .

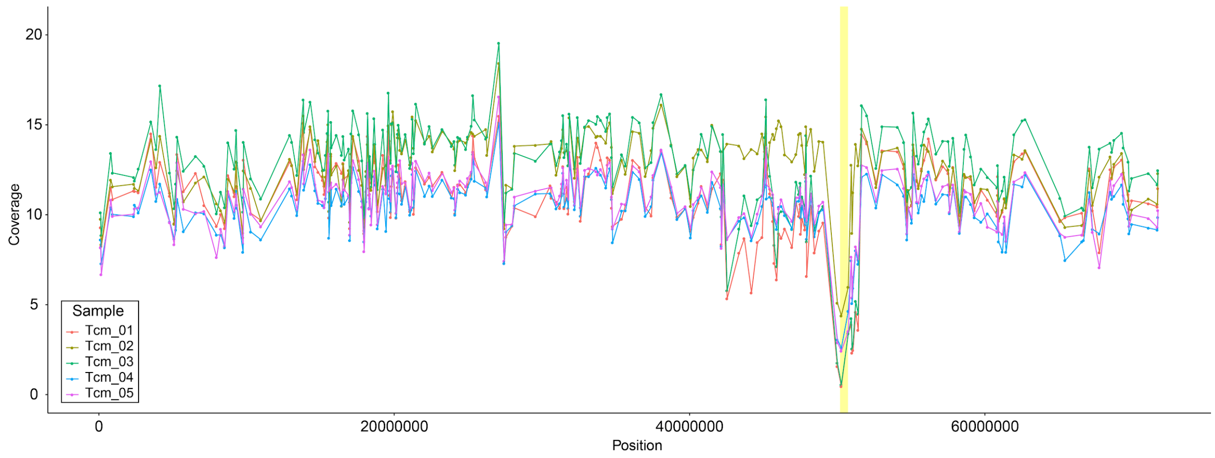

Fig. S12. Coverage along LG8 for 5 resequenced *T. californicum*. Coverage was estimated by mapping reads to the *T. californicum* genome scaffolds, and scaffolds were anchored on *T. cristinae* linkage groups (see Methods and Supplementary Text). The region with the expected deletion is highlighted in yellow. If there was a deletion on LG8 determining the green morph, grey individual Tcm_02 should feature higher coverage than the other individuals which are green.

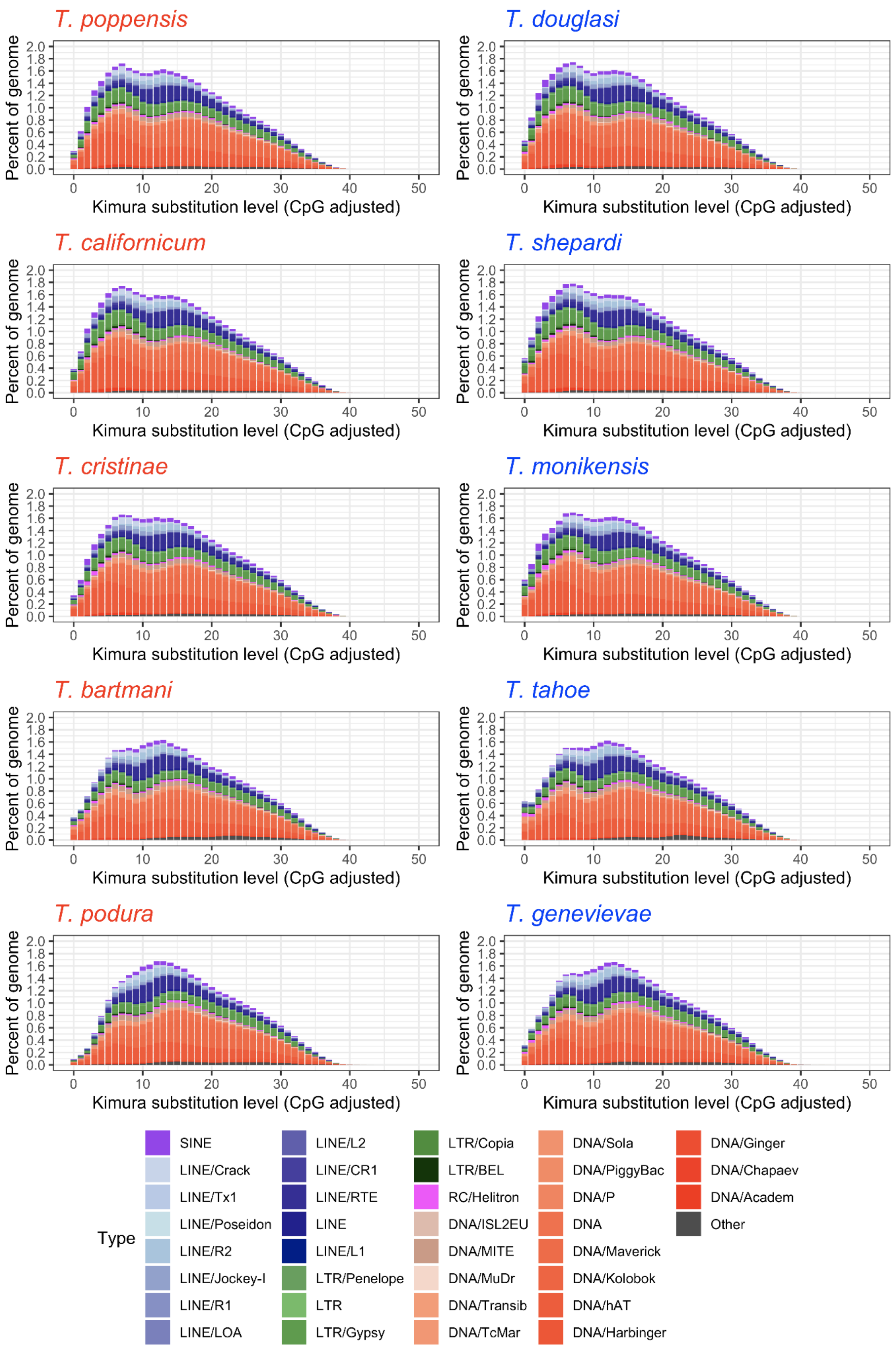

Fig. S13. TE landscapes indicate recent TE activity is low. Kimura substitution level is a measure of divergence of the TE copies to the consensus TE sequence. Recent TE expansions would cause high peaks close to 0. Note that the plots for

closely related *Timema* species are very similar (i.e., the abundance peaks are at similar divergence levels), reflecting the TE activity in their common ancestors. Also note that although TE copies with small divergence levels (up to ~3%) appear to be more abundant in parthenogenetic than sexual species, this is likely an artefact caused by the systematically better genome assembly quality in the parthenogenetic species (see main text and Fig. S14), with similar TE copies more often collapsed in the assemblies of sexual than parthenogenetic species.

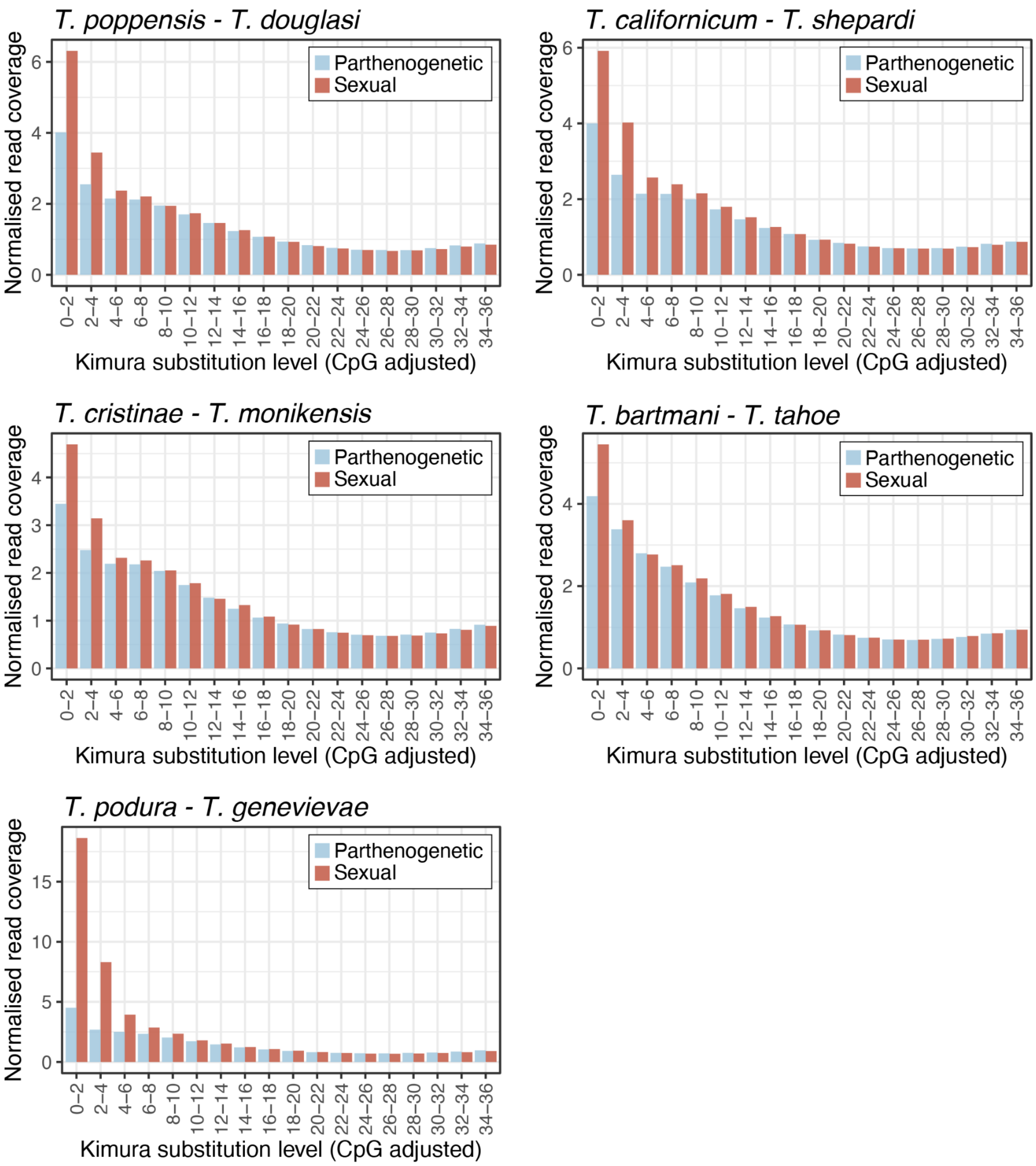

Fig. S14. Coverage of TE landscapes shows TE copies with low divergence from the consensus TE sequence (measured as Kimura substitution level) is higher for sexual species than parthenogenetic species. This indicates that similar TE copies are more often collapsed in the assemblies of sexual than parthenogenetic species. Coverage was normalised by the median TE coverage for each species separately.

Table S1. Origin of biological material

All six females per species were taken from a single location at the indicated coordinates. Reproductive mode (Rep. mode) is indicated by an S for sexual species or a P for parthenogenetic species.

Species	Rep. mode	Host plant	Coordinates	
			longitude	latitude
T. tahoe	P	*Abies concolor*	38.7610110	-120.1600530
T. bartmani	S	*Abies concolor*	34.1700000	-117.0020167
T. shepardi	P	*Arctostaphylos sp.*	39.1926500	-123.2617833
T. californicum	S	*Quercus sp.*	37.3431667	-121.6364667
T. douglasi	P	*Pseudotsuga menziesii*	38.9825500	-123.4697500
T. poppensis	S	*Sequoia sempervirens*	37.1655167	-122.0155500
T. monikensis	P	*Cercocarpus betuloides*	34.1148833	-118.8531333
T. cristinae	S	*Cercocarpus betuloides*	34.5362700	-119.2444300
T. genevievae	P	*Adenostoma fasciculatum*	38.9957833	-122.9257667
T. podura	S	*Adenostoma fasciculatum*	33.7976020	-116.7769850

Table S2. Sequencing coverage

Read coverage for the reference assemblies of individual *Timema* species was estimated using the haploid genome size of *Timema cristinae* of 1.381 Gbp (*20*). Is: insert size [bp].

Species	Paired-end			Mate-pair		Orphans	Total
	Is 350	Is 550	Is 700	Is 3000	Is 5000		
T. tahoe	15	12.2	5.7	4.1	3	3	43.1
T. bartmani	12.3	13.5	3.7	2.7	2.5	2.4	37.0
T. shepardi	12.4	11.6	8.3	3.8	3.6	2.8	42.7
T.californicum	16.4	13.2	8.1	4.4	2.8	3.1	48.2
T. douglasi	13.2	11	8.8	4.3	2.8	2.9	43.1
T. poppensis	12.5	12.1	7.1	2.9	2.8	2.7	40.2
T. monikensis	13.8	12.6	9.6	3.4	4.2	3	46.6
T. cristinae	13.7	10.9	10	4	3.6	3	45.3
T. genevievae	15	13.4	4.3	2.5	5.4	2.8	43.5
T. podura	15.7	10.8	3.1	3.1	2.6	2.3	37.7

Table S3. Genome assembly statistics

Genome assembly statistics of sequenced *Timema* species. Haploid genome size represents the estimate from genome profiling of raw reads using Genomescope (*22*). Total sum represents the sum of all scaffolds. The BUSCO score (*21*) is the percentage of conserved single copy orthologs among insects. N is the percentage of unknown nucleotides (gaps) in the assembly. Genes are the number of annotated protein coding genes. Reproductive mode (Rep. mode) is indicated by either an S for sexual species or a P for parthenogenetic species. Although the sequencing coverage was similar across the ten sequenced species (approximately 40x, Table S2), all five parthenogenetic species had both higher continuity (N50 96.3 - 226.5 kbp for parthenogens, vs. 4.1 - 112.7 kbp for sexuals) and higher completeness (97.2 - 98.3% BUSCO genes in parthenogens, vs. 86.4 - 97.2% in sexuals), likely because of systematic differences in heterozygosity between species with different reproductive modes (see main text).

Species	Rep. mode	Haploid genome size [Gpb]	Σ [Gpb]	N50 [kbp]	BUSCO [%]	Ns [%]	Genes
T. tahoe	P	1.13	1.093	125.4	97.5	2.4	12771
T. bartmani	S	1.15	1.109	104.8	97.2	2.6	14066
T. shepardi	P	1.23	1.153	103.5	97.2	1.7	14033
T.californicum	S	1.3	1.220	66.6	94.4	1.8	14563
T. douglasi	P	1.26	1.124	96.3	97.2	1.6	13824
T. poppensis	S	1.31	1.137	39.6	93.9	1.6	15605
T. monikensis	P	1.12	1.099	226.5	98.3	1.7	12837
T. cristinae	S	1.11	1.178	112.7	96.9	2.3	13882
T. genevievae	P	1.07	1.049	141.4	979	1.6	12009
T. podura	S	1.04	1.105	4.09	86.4	0.4	16529

Table S4. Origin of the genetic variation among genotypes in parthenogenetic populations

To distinguish between putative ancestral polymorphisms (shared between sexual and parthenogenetic species) and polymorphisms that appeared in the parthenogenetic lineage after the split from the sexual lineage, we used the SNPs generated for heterozygosity estimates via GATK best practices pipeline (*62*) (see Methods) but with less stringent downstream filtering (min 10x coverage). Homologous SNPs within a species pair were identified with MUMmer v4.0.0beta2 (nucmer and dnadiff with default parameters to keep only unique alignments of genome segments, and custom scripts to discard overlapping alignments), using the genome of the parthenogenetic species as the reference and the one from its sexual relative as the query.

Species pair	Number of positions analyzed	Variable (within and/or between species)	Same variants in both species	Different variants	Variable only in sexual species	Variable only in partheno-genetic species
T. bartmani *T. tahoe*	852224058	8945655	26137	3683201	5052559	183758
T. californicum *T. shepardi*	725333178	12631427	51243	4752391	7604702	223091
T. cristinae *T. monikensis*	816642553	19793873	87370	7078329	11677904	950270
T. poppensis *T. douglasi*	781906596	14109700	206188	8296511	3989068	1617933
T. podura *T. genevievae*	636577084	27365408	325	5947476	21410167	7440

Table S5. GO terms enriched in positively selected genes

Few GO terms are enriched in positively selected genes in each species. This may be partly due to the difficulty in obtaining functional annotations in *Timema*, due to their evolutionary distance from a well characterised insect model system. Species are abbreviated as follows: Tbi = *T. bartmani*, Tce = *T. cristinae*, Tps = *T. poppensis*, Tcm = *T. californicum*, Tpa = *T. podura*, Tte = *T. tahoe*, Tms = *T. monikensis*, Tdi = *T. douglasi*, Tsi = *T. shepardi*, and Tge = *T. genevievae*

GO ID	Term	Annotated	Significant	Expected	p	sp
GO:0007399	nervous system development	315	12	5.08	0.0028	Tte
GO:0006338	chromatin remodeling	20	3	0.32	0.0035	Tte
GO:0007476	imaginal disc-derived wing morphogenesis	45	4	0.73	0.0055	Tte
GO:0050775	positive regulation of dendrite morphogenesis	30	3	0.48	0.0118	Tte
GO:0030178	negative regulation of Wnt signaling pathway	31	3	0.5	0.0129	Tte
GO:0043039	tRNA aminoacylation	12	2	0.19	0.0152	Tte
GO:0031935	regulation of chromatin silencing	14	2	0.23	0.0205	Tte
GO:0008593	regulation of Notch signaling pathway	14	2	0.23	0.0205	Tte
GO:0045931	positive regulation of mitotic cell cycle	15	2	0.24	0.0234	Tte
GO:0006030	chitin metabolic process	17	2	0.27	0.0297	Tte
GO:0009058	biosynthetic process	454	10	7.33	0.0306	Tte
GO:0060966	regulation of gene silencing by RNA	16	2	0.26	0.0315	Tte
GO:0046331	lateral inhibition	44	3	0.71	0.0329	Tte
GO:0007155	cell adhesion	47	3	0.76	0.0389	Tte
GO:0007286	spermatid development	20	2	0.32	0.0403	Tte
GO:0006997	nucleus organization	20	2	0.32	0.0403	Tte
GO:0032990	cell part morphogenesis	127	7	2.05	0.0435	Tte
GO:0048814	regulation of dendrite morphogenesis	33	4	0.53	0.0451	Tte
GO:0002064	epithelial cell development	84	4	1.36	0.0459	Tte
GO:0032259	methylation	30	3	0.33	0.0011	Tbi
GO:0007631	feeding behavior	12	2	0.13	0.007	Tms
GO:0007450	dorsal/ventral pattern formation, imaginal disc	12	2	0.13	0.007	Tms
GO:0016485	protein processing	20	2	0.22	0.019	Tms
GO:0007601	visual perception	22	2	0.24	0.023	Tms
GO:0007088	regulation of mitotic nuclear division	22	2	0.24	0.023	Tms
GO:0031667	response to nutrient levels	29	3	0.31	0.03	Tms
GO:0007623	circadian rhythm	29	2	0.31	0.039	Tms
GO:0110116	regulation of compound eye photoreceptor cell differentiation	31	3	0.44	0.0019	Tsi
GO:0045732	positive regulation of protein catabolic process	10	2	0.14	0.0084	Tsi
GO:0043269	regulation of ion transport	12	2	0.17	0.0121	Tsi
GO:0031331	positive regulation of cellular catabolic process	15	2	0.21	0.0187	Tsi
GO:0045466	R7 cell differentiation	17	2	0.24	0.0237	Tsi
GO:0016197	endosomal transport	17	2	0.24	0.0237	Tsi

GO:0044773	mitotic DNA damage checkpoint	19	2	0.27	0.0279	Tsi
GO:0035088	establishment or maintenance of apical/basal cell polarity	21	2	0.3	0.0354	Tsi
GO:0006520	cellular amino acid metabolic process	32	2	0.46	0.0418	Tsi
GO:0006261	DNA-dependent DNA replication	13	2	0.18	0.013	Tcm
GO:0098869	cellular oxidant detoxification	14	2	0.2	0.016	Tcm
GO:1903008	organelle disassembly	14	2	0.2	0.016	Tcm
GO:0007052	mitotic spindle organization	14	2	0.2	0.016	Tcm
GO:0003007	heart morphogenesis	10	2	0.2	0.016	Tdi
GO:0071985	multivesicular body sorting pathway	10	2	0.2	0.016	Tdi
GO:0044262	cellular carbohydrate metabolic process	42	3	0.84	0.03	Tdi
GO:0033206	meiotic cytokinesis	15	2	0.3	0.039	Tdi
GO:0051046	regulation of secretion	21	2	0.42	0.039	Tdi
GO:0072657	protein localization to membrane	22	2	0.44	0.039	Tdi
GO:0098656	anion transmembrane transport	16	2	0.32	0.039	Tdi
GO:0030001	metal ion transport	17	2	0.34	0.044	Tdi
GO:0016197	endosomal transport	34	3	0.68	0.048	Tdi
GO:0030855	epithelial cell differentiation	81	2	0.57	0.027	Tge
GO:0007030	Golgi organization	50	2	0.35	0.046	Tge
GO:0010499	proteasomal ubiquitin-independent protein catabolic process	13	2	0.18	0.013	Tpa
GO:0001510	RNA methylation	14	2	0.19	0.015	Tpa

Table S6. Accession numbers for raw reads of reference individuals

Asterisks indicate which liberty was used for each analysis. Species are abbreviated as follows: Tbi = *T. bartmani*, Tce = *T. cristinae*, Tps = *T. poppensis*, Tcm = *T. californicum*, Tpa = *T. podura*, Tte = *T. tahoe*, Tms = *T. monikensis*, Tdi = *T. douglasi*, Tsi = *T. shepardi*, and Tge = *T. genevievae*

Library Name	Sp ID	Sample ID	Insert size	SRA sample accession	SRA run accession	Assembly	Genome profiling	Variants
HYI-7_125	4_Tte	Tte_00	350	SRS1972401	SRR5248900	*	*	
HYI-7_150	4_Tte	Tte_00	350	SRS1972401	SRR5248899		*	
HYI-17	4_Tte	Tte_00	550	SRS1972401	SRR5248898	*	*	*
HYI-51	4_Tte	Tte_00	700	SRS1972401	SRR5248897	*	*	
HYI-37	4_Tte	Tte_00	3000	SRS1972401	SRR5248896	*		
HYI-47	4_Tte	Tte_00	5000	SRS1972401	SRR5248895	*		
HYI-18_125	4_Tbi	Tbi_00	350	SRS1972400	SRR5248892	*	*	
HYI-8_125	4_Tbi	Tbi_00	350	SRS1972400	SRR5248894		*	
HYI-8_150	4_Tbi	Tbi_00	350	SRS1972400	SRR5248893	*	*	*
HYI-28	4_Tbi	Tbi_00	700	SRS1972400	SRR5248891	*	*	
HYI-38	4_Tbi	Tbi_00	3000	SRS1972400	SRR5248890	*		
HYI-48	4_Tbi	Tbi_00	5000	SRS1972400	SRR5248889	*		
HYI-4_125	2_Tsi	Tsi_00	350	SRS1972405	SRR5248924	*	*	
HYI-4_150	2_Tsi	Tsi_00	350	SRS1972405	SRR5248923		*	
HYI-14	2_Tsi	Tsi_00	550	SRS1972405	SRR5248922	*	*	*
HYI-24	2_Tsi	Tsi_00	700	SRS1972405	SRR5248921	*	*	
HYI-34	2_Tsi	Tsi_00	3000	SRS1972405	SRR5248920	*		
HYI-44	2_Tsi	Tsi_00	5000	SRS1972405	SRR5248919	*		
HYI-3_125	2_Tcm	Tcm_00	350	SRS1972404	SRR5248918	*	*	
HYI-3_150	2_Tcm	Tcm_00	350	SRS1972404	SRR5248917		*	
HYI-13	2_Tcm	Tcm_00	550	SRS1972404	SRR5248916	*	*	*
HYI-23	2_Tcm	Tcm_00	700	SRS1972404	SRR5248915	*	*	
HYI-33	2_Tcm	Tcm_00	3000	SRS1972404	SRR5248914	*		
HYI-43	2_Tcm	Tcm_00	5000	SRS1972404	SRR5248913	*		
HYI-5_125	3_Tms	Tms_00	350	SRS1972403	SRR5248912	*	*	
HYI-5_150	3_Tms	Tms_00	350	SRS1972403	SRR5248911		*	
HYI-15	3_Tms	Tms_00	550	SRS1972403	SRR5248910	*	*	*
HYI-25	3_Tms	Tms_00	700	SRS1972403	SRR5248909	*	*	
HYI-35	3_Tms	Tms_00	3000	SRS1972403	SRR5248908	*		
HYI-45	3_Tms	Tms_00	5000	SRS1972403	SRR5248907	*		
HYI-6_125	3_Tce	Tce_00	350	SRS1972402	SRR5248906	*	*	
HYI-6_150	3_Tce	Tce_00	350	SRS1972402	SRR5248905		*	
HYI-16	3_Tce	Tce_00	550	SRS1972402	SRR5248904	*	*	*
HYI-26	3_Tce	Tce_00	700	SRS1972402	SRR5248903	*	*	
HYI-36	3_Tce	Tce_00	3000	SRS1972402	SRR5248902	*		
HYI-46	3_Tce	Tce_00	5000	SRS1972402	SRR5248901	*		
HYI-1_125	1_Tdi	Tdi_00	350	SRS1972407	SRR5248936	*	*	
HYI-1_150	1_Tdi	Tdi_00	350	SRS1972407	SRR5248935		*	
HYI-11	1_Tdi	Tdi_00	550	SRS1972407	SRR5248934	*	*	*
HYI-21	1_Tdi	Tdi_00	700	SRS1972407	SRR5248933	*	*	

HYI-31	1_Tdi	Tdi_00	3000	SRS1972407	SRR5248932	*		
HYI-41	1_Tdi	Tdi_00	5000	SRS1972407	SRR5248931	*		
HYI-2_125	1_Tps	Tps_00	350	SRS1972406	SRR5248930	*	*	
HYI-2_150	1_Tps	Tps_00	350	SRS1972406	SRR5248929		*	
HYI-12	1_Tps	Tps_00	550	SRS1972406	SRR5248928	*	*	*
HYI-22	1_Tps	Tps_00	700	SRS1972406	SRR5248927	*	*	
HYI-32	1_Tps	Tps_00	3000	SRS1972406	SRR5248926	*		
HYI-42	1_Tps	Tps_00	5000	SRS1972406	SRR5248925	*		
HYI-10_125	5_Tge	Tge_00	350	SRS1972399	SRR5248888	*	*	
HYI-10_150	5_Tge	Tge_00	350	SRS1972399	SRR5248887		*	
HYI-20	5_Tge	Tge_00	550	SRS1972399	SRR5248886	*	*	*
HYI-53	5_Tge	Tge_00	700	SRS1972399	SRR5248885	*	*	
HYI-40	5_Tge	Tge_00	3000	SRS1972399	SRR5248884	*		
HYI-50	5_Tge	Tge_00	5000	SRS1972399	SRR5248883	*		
HYI-9_125	5_Tpa	Tpa_00	350	SRS1972398	SRR5248882	*	*	
HYI-9_150	5_Tpa	Tpa_00	350	SRS1972398	SRR5248881		*	
HYI-19	5_Tpa	Tpa_00	550	SRS1972398	SRR5248880	*	*	*
HYI-52	5_Tpa	Tpa_00	700	SRS1972398	SRR5248879	*	*	
HYI-39	5_Tpa	Tpa_00	3000	SRS1972398	SRR5248878	*		
HYI-49	5_Tpa	Tpa_00	5000	SRS1972398	SRR5248877	*		

Table S7. Accession numbers for raw reads of resequenced individuals

Species are abbreviated as follows: Tbi = *T. bartmani*, Tce = *T. cristinae*, Tps = *T. poppensis*, Tcm = *T. californicum*, Tpa = *T. podura*, Tte = *T. tahoe*, Tms = *T. monikensis*, Tdi = *T. douglasi*, Tsi = *T. shepardi*, and Tge = *T. genevievae*

Library Name	Species ID	Sample ID	SRA sample accession	SRA run accession
ReSeq_Te07	4_Tte	Tte_01	SRS7638306	SRR12928425, SRR12928426, SRR12928429-SRR12928438, SRR12928440-SRR12928449
ReSeq_Te08	4_Tte	Tte_02	SRS7638305	SRR12928399-SRR12928404, SRR12928406-SRR12928415, SRR12928417-SRR12928424
ReSeq_Te09	4_Tte	Tte_03	SRS7638326	SRR12928367-SRR12928371, SRR12928373-SRR12928382, SRR12928384-SRR12928393, SRR12928395-SRR12928398
ReSeq_Te10	4_Tte	Tte_04	SRS7638327	SRR12928340-SRR12928349, SRR12928351-SRR12928360, SRR12928362-SRR12928366
ReSeq_Te11	4_Tte	Tte_05	SRS7638328	SRR12928311-SRR12928315, SRR12928318-SRR12928327, SRR12928329-SRR12928338
CC86B	4_Tbi	Tbi_01	SRS7637496	SRR12928843-SRR12928847, SRR12928849-SRR12928858, SRR12928860
CC86C	4_Tbi	Tbi_02	SRS7637495	SRR12928821-SRR12928824, SRR12928826-SRR12928835, SRR12928838-SRR12928842
CC87B	4_Tbi	Tbi_03	SRS7637498	SRR12928490-SRR12928493, SRR12928495-SRR12928504, SRR12928506-SRR12928515, SRR12928517-SRR12928520, SRR12928818, SRR12928820

CC87C	4_Tbi	Tbi_04	SRS7638307	SRR12928468-SRR12928471, SRR12928473-SRR12928482, SRR12928484-SRR12928489
CC88B	4_Tbi	Tbi_05	SRS7638309	SRR12928451-SRR12928460, SRR12928462-SRR12928467
ReSeq_Si01	2_Tsi	Tsi_01	SRS7638289	SRR12928651-SRR12928659, SRR12928661-SRR12928663
ReSeq_S14	2_Tsi	Tsi_02	SRS7638288	SRR12928664-SRR12928670, SRR12928672-SRR12928676
ReSeq_Si03	2_Tsi	Tsi_03	SRS7638287	SRR12928635-SRR12928637, SRR12928639-SRR12928648, SRR12928650
ReSeq_Si16	2_Tsi	Tsi_04	SRS7638284	SRR12928621-SRR12928626, SRR12928628-SRR12928634
ReSeq_Si18	2_Tsi	Tsi_05	SRS7638285	SRR12928604, SRR12928606-SRR12928615, SRR12928617-SRR12928620
HM217	2_Tcm	Tcm_01	SRS7638279	SRR12928757-SRR12928759, SRR12928761-SRR12928770, SRR12928772-SRR12928778
HM218	2_Tcm	Tcm_02	SRS7638277	SRR12928735-SRR12928737, SRR12928739-SRR12928748, SRR12928750-SRR12928756
HM219	2_Tcm	Tcm_03	SRS7638281	SRR12928713-SRR12928715, SRR12928717-SRR12928726, SRR12928728-SRR12928734
HM220	2_Tcm	Tcm_04	SRS7638282	SRR12928695-SRR12928703, SRR12928706-SRR12928712
HM221	2_Tcm	Tcm_05	SRS7638286	SRR12928677-SRR12928681, SRR12928683-SRR12928692, SRR12928694

ReSeq_Ms01	3_Tms	Tms_01	SRS7637486	SRR12928998-SRR12929002, SRR12929004-SRR12929013, SRR12929015
ReSeq_Ms02	3_Tms	Tms_02	SRS7637485	SRR12928916, SRR12928918-SRR12928924, SRR12928988, SRR12928990, SRR12928991, SRR12928993-SRR12928997
ReSeq_Ms03	3_Tms	Tms_03	SRS7637493	SRR12928896, SRR12928898-SRR12928905, SRR12928907-SRR12928915
MS_Alp03b	3_Tms	Tms_04	SRS7637467	SRR12929069-SRR12929077, SRR12929080-SRR12929089, SRR12929091, SRR12929092
MS_Alp04b	3_Tms	Tms_05	SRS7637463	SRR12929016, SRR12929048-SRR12929055, SRR12929057-SRR12929066, SRR12929068
CC22B	3_Tce	Tce_01	SRS7638290	SRR12928577-SRR12928581, SRR12928583-SRR12928592, SRR12928595-SRR12928603
CC22C	3_Tce	Tce_02	SRS7638291	SRR12928555-SRR12928559, SRR12928561-SRR12928570, SRR12928572-SRR12928576
CC24B	3_Tce	Tce_03	SRS7638292	SRR12928533-SRR12928537, SRR12928539-SRR12928548, SRR12928550-SRR12928554
CC24C	3_Tce	Tce_04	SRS7637466	SRR12928521-SRR12928526, SRR12928528-SRR12928532, SRR12928819, SRR12929111, SRR12929113-SRR12929115
CC25B	3_Tce	Tce_05	SRS7637461	SRR12929093-SRR12929100, SRR12929102-SRR12929110

ReSeq_Di02	1_Tdi	Tdi_01	SRS7637469	SRR12928239, SRR12928250, SRR12928261, SRR12928272, SRR12928283, SRR12928294, SRR12928305, SRR12928961, SRR12928972, SRR12928983, SRR12929022, SRR12929034, SRR12929045
ReSeq_Di04	1_Tdi	Tdi_02	SRS7637489	SRR12928865-SRR12928870, SRR12928872, SRR12928878, SRR12928889, SRR12928928, SRR12928939, SRR12928950
ReSeq_Di06	1_Tdi	Tdi_03	SRS7637497	SRR12928806-SRR12928814, SRR12928861-SRR12928864
ReSeq_Di08	1_Tdi	Tdi_04	SRS7638280	SRR12928792, SRR12928794-SRR12928803, SRR12928805
ReSeq_Di10	1_Tdi	Tdi_05	SRS7638278	SRR12928779-SRR12928781, SRR12928783-SRR12928791
ReSeq_Ps14	1_Tps	Tps_01	SRS7637462	SRR12928527, SRR12928538, SRR12928549, SRR12928560, SRR12928571, SRR12928582, SRR12928593, SRR12928605, SRR12929014, SRR12929056, SRR12929067, SRR12929078, SRR12929090, SRR12929101, SRR12929112
ReSeq_Ps16	1_Tps	Tps_02	SRS7637490	SRR12928483, SRR12928494, SRR12928505, SRR12928516, SRR12928825, SRR12928836, SRR12928848, SRR12928859, SRR12928906, SRR12928917, SRR12928992, SRR12929003

ReSeq_Ps18	1_Tps	Tps_03	SRS7638308	SRR12928316, SRR12928328, SRR12928339, SRR12928350, SRR12928361, SRR12928372, SRR12928383, SRR12928394, SRR12928405, SRR12928416, SRR12928427, SRR12928439, SRR12928450, SRR12928461, SRR12928472
ReSeq_Ps08	1_Tps	Tps_04	SRS7637470	SRR12928317, SRR12928428, SRR12928594, SRR12928705, SRR12928782, SRR12928793, SRR12928804, SRR12928815-SRR12928817, SRR12928837, SRR12928871, SRR12929023, SRR12929079
ReSeq_Ps12	1_Tps	Tps_05	SRS7638283	SRR12928616, SRR12928627, SRR12928638, SRR12928649, SRR12928660, SRR12928671, SRR12928682, SRR12928693, SRR12928704, SRR12928716, SRR12928727, SRR12928738, SRR12928749, SRR12928760, SRR12928771
CC59_A	5_Tge	Tge_01	SRS7637468	SRR12928980-SRR12928982, SRR12928984-SRR12928987, SRR12928989, SRR12929017-SRR12929021, SRR12929024-SRR12929026
CC59_C	5_Tge	Tge_02	SRS7637484	SRR12928958-SRR12928960, SRR12928962-SRR12928971, SRR12928973-SRR12928979
CC65_B	5_Tge	Tge_03	SRS7637488	SRR12928937, SRR12928938, SRR12928940-SRR12928949, SRR12928951-SRR12928957

CC66_A	5_Tge	Tge_04	SRS7637487	SRR12928892-SRR12928895, SRR12928897, SRR12928925-SRR12928927, SRR12928929-SRR12928936
CC67_A	5_Tge	Tge_05	SRS7637494	SRR12928873-SRR12928877, SRR12928879-SRR12928888, SRR12928890, SRR12928891
Pa_AB	5_Tpa	Tpa_01	SRS7637465	SRR12929027-SRR12929033, SRR12929035-SRR12929043
PA_CD	5_Tpa	Tpa_02	SRS7638329	SRR12928245-SRR12928249, SRR12928251-SRR12928260, SRR12928262, SRR12928263
PA_E	5_Tpa	Tpa_03	SRS7637464	SRR12928231-SRR12928238, SRR12928240-SRR12928244, SRR12929044, SRR12929046, SRR12929047
H54	5_Tpa	Tpa_04	SRS7638331	SRR12928293, SRR12928295-SRR12928304, SRR12928306-SRR12928310
H56	5_Tpa	Tpa_05	SRS7638330	SRR12928264-SRR12928271, SRR12928273-SRR12928282, SRR12928284-SRR12928292

Table S8. Number of RNA-seq libraries used for genome annotation

Species are abbreviated as follows: Tbi = *T. bartmani*, Tce = *T. cristinae*, Tps = *T. poppensis*, Tcm = *T. californicum*, Tpa = *T. podura*, Tte = *T. tahoe*, Tms = *T. monikensis*, Tdi = *T. douglasi*, Tsi = *T. shepardi*, and Tge = *T. genevievae*. Bioproject Accessions: PRJNA679785, PRJNA678950, PRJNA380865, PRJNA392384

Tissue	Library type	Tbi	Tte	Tce	Tms	Tcm	Tsi	Tpa	Tge	Tps	Tdi
Whole-Body (Female)	Single-end	6	3	6	3	6	3	6	3	6	3
Whole-Body (Male)	Single-end	3		3		3		3		3	
Rep. tract (Female)	Single-end	3	3	3	3	3	3	3	3	3	3
Rep. tract (Male)	Single-end	3		3		3		3		3	
Heads (Female)	Single-end	3	3	3	3	3	3	3	3	3	3
Heads (Male)	Single-end	3		3		3		3		3	
Legs (Female)	Single-end	3	3	3	3	3	3	3	3	3	3
Legs (Male)	Single-end	3		3		3		3		3	
Juvenile (Female)	Paired-end					3	3				
Juvenile (Male)	Paired-end					3					
Hatchling (Unknown)	Paired-end			7	3	6	3	5			3

Table S9. Microsatellites located in the v1.3 genome of *T. cristinae*. Msat name: Microsatellite names from (*17*). Indicated are the scaffolds where a given microsatellite was found (Scaffold), the location of the microsatellite midpoint on the scaffold (Position), the linkage group (LG), the size of the microsatellite in the v1.3 assembly and the expected size range given microsatellite genotypes in *T. cristinae* (Length (expected)), and whether the expected microsatellite repeat motif was present.

Msat name	Scaffold	LG	Position	Length (expected) [bp]	Motif
**tim-3 (a)**	CM009483.1	LG8	32962807	159 (82-109)	Yes
**tim-3 (b)**	CM009476.1	LG13	19984463	169 (82-109)	Yes
**tim-4**	CM009477.1	LG2	46600506	119 (83-125)	Yes
**tim-5**	CM009481.1	LG6	7421031	198 (124-238)	Yes
**tim-6**	CM009474.1	LG11	12218418	264 (253-283)	Yes
**tim-7**	CM009482.1	LG7	19035853	156 (120-195)	Yes
**tim-8 (a)**	CM009484.1	LG9	23487739	138 (133-148)	Yes
**tim-8 (b)**	CM009479.1	LG4	85533694	190 (133-148)	No
**tim-8 (c)**	CM009478.1	LG3	981498	498 (133-148)	No

REFERENCES AND NOTES

1. M. Neiman, C. M. Lively, S. Meirmans, Why sex? A pluralist approach revisited. *Trends Ecol. Evol.* **32**, 589–600 (2017).

2. N. P. Sharp, S. P. Otto, Evolution of sex: Using experimental genomics to select among competing theories. *Bioessays* **38**, 751–757 (2016).

3. G. Bell, *The Masterpiece of Nature: The Evolution and Genetics of Sexuality* (University of California Press, 1982).

4. G. C. Williams, *Sex and Evolution* (Princeton Univ. Press, 1975).

5. J. Maynard Smith, *The Evolution of Sex* (Cambridge Univ. Press, 1978).

6. J. Gerritsen, Sex and parthenogenesis in sparse populations. *Am. Nat.* **115**, 718–742 (1980).

7. S. K. Jain, The evolution of inbreeding in plants. *Annu. Rev. Ecol. Syst.* **7**, 469–495 (1976).

8. J. Felsenstein, The evolutionary advantage of recombination. *Genetics* **78**, 737–756 (1974).

9. W. G. Hill, A. Robertson, The effect of linkage on limits to artificial selection. *Genet. Res.* **8**, 269–294 (1966).

10. P. D. Keightley, S. P. Otto, Interference among deleterious mutations favours sex and recombination in finite populations. *Nature* **443**, 89–92 (2006).

11. N. H. Barton, Why sex and recombination? *Cold Spring Harb. Symp. Quant. Biol.* **74**, 187–195 (2009).

12. C. W. Birky Jr, Heterozygosity, heteromorphy, and phylogenetic trees in asexual eukaryotes. *Genetics* **144**, 427–437 (1996).

13. D. Mark Welch, M. Meselson, Evidence for the evolution of bdelloid rotifers without sexual reproduction or genetic exchange. *Science* **288**, 1211–1215 (2000).

14. D. A. Hickey, Selfish DNA: A sexually-transmitted nuclear parasite. *Genetics* **101**, 519–531 (1982).

15. E. S. Dolgin, B. Charlesworth, The effects of recombination rate on the distribution and abundance of transposable elements. *Genetics* **178**, 2169–2177 (2008).

16. K. S. Jaron, J. Bast, R. W. Nowell, T. R. Ranallo-Benavidez, M. Robinson-Rechavi, T. Schwander, Genomic features of parthenogenetic animals. *J. Hered.* **112**, 19–33 (2021).

17. T. Schwander, B. J. Crespi, Multiple direct transitions from sexual reproduction to apomictic parthenogenesis in *Timema* stick insects. *Evolution* **63**, 84–103 (2009).

18. V. Soria-Carrasco, Z. Gompert, A. A. Comeault, T. E. Farkas, T. L. Parchman, J. S. Johnston, C. A. Buerkle, J. L. Feder, J. Bast, T. Schwander, S. P. Egan, B. J. Crespi, P. Nosil, Stick insect genomes reveal natural selection's role in parallel speciation. *Science* **344**, 738–742 (2014).

19. R. M. Waterhouse, M. Seppey, F. A. Simão, M. Manni, P. Ioannidis, G. Klioutchnikov, E. V. Kriventseva, E. M. Zdobnov, BUSCO applications from quality assessments to gene prediction and phylogenomics. *Mol. Biol. Evol.* **35**, 543–548 (2018).

20. T. R. Ranallo-Benavidez, K. S. Jaron, M. C. Schatz, GenomeScope 2.0 and Smudgeplot for reference-free profiling of polyploid genomes. *Nat. Commun.* **11**, 1432 (2020).

21. J. Romiguier, P. Gayral, M. Ballenghien, A. Bernard, V. Cahais, A. Chenuil, Y. Chiari, R. Dernat, L. Duret, N. Faivre, E. Loire, J. M. Lourenco, B. Nabholz, C. Roux, G. Tsagkogeorga, A. A.-T. Weber, L. A. Weinert, K. Belkhir, N. Bierne, S. Glémin, N. Galtier, Comparative population genomics in animals uncovers the determinants of genetic diversity. *Nature* **515**, 261–263 (2014).

22. T. Schwander, S. Vuilleumier, J. Dubman, B. J. Crespi, Positive feedback in the transition from sexual reproduction to parthenogenesis. *Proc. Biol. Sci.* **277**, 1435–1442 (2010).

23. J. Engelstädter, Asexual but not clonal: Evolutionary processes in automictic populations. *Genetics* **206**, 993–1009 (2017).

24. M. Neiman, T. Schwander, Using parthenogenetic lineages to identify advantages of sex. *Evol. Biol.* **38**, 115–123 (2011).

25. S. Glémin, C. M. François, N. Galtier, Genome evolution in outcrossing vs. selfing vs. asexual species. *Methods Mol. Biol.* **1910**, 331–369 (2019).

26. M. Pearcy, S. Aron, C. Doums, L. Keller, Conditional use of sex and parthenogenesis for worker and queen production in ants. *Science* **306**, 1780–1783 (2004).

27. M. O. Lorenzo-Carballa, A. Cordero-Rivera, Thelytokous parthenogenesis in the damselfly Ischnura hastata (Odonata, Coenagrionidae): Genetic mechanisms and lack of bacterial infection. *Heredity* **103**, 377–384 (2009).

28. T. J. Treangen, S. L. Salzberg, Repetitive DNA and next-generation sequencing: Computational challenges and solutions. *Nat. Rev. Genet.* **13**, 36–46 (2011).

29. H. Ellegren, N. Galtier, Determinants of genetic diversity. *Nat. Rev. Genet.* **17**, 422–433 (2016).

30. P. Nosil, R. Villoutreix, C. F. de Carvalho, T. E. Farkas, V. Soria-Carrasco, J. L. Feder, B. J. Crespi, Z. Gompert, Natural selection and the predictability of evolution in *Timema* stick insects. *Science* **359**, 765–770 (2018).

31. T. Schwander, L. Henry, B. J. Crespi, Molecular evidence for ancient asexuality in *Timema* stick insects. *Curr. Biol.* **21**, 1129–1134 (2011).

32. V. R. Vickery, Revision *of Timema* scudder (Phasmatoptera: Timematodea) including three new species. *Can. Entomol.* **125**, 657–692 (1993).

33. J. Bast, D. J. Parker, Z. Dumas, K. M. Jalvingh, P. Tran Van, K. S. Jaron, E. Figuet, A. Brandt, N. Galtier, T. Schwander, Consequences of asexuality in natural populations: Insights from stick insects. *Mol. Biol. Evol.* **35**, 1668–1677 (2018).

34. L. Henry, T. Schwander, B. J. Crespi, Deleterious mutation accumulation in asexual Timema stick insects. *Mol. Biol. Evol.* **29**, 401–408 (2012).

35. M. Neiman, P. G. Meirmans, T. Schwander, S. Meirmans, Sex in the wild: How and why field-based studies contribute to solving the problem of sex. *Evolution* **72**, 1194–1203 (2018).

36. S. P. Otto, Selective interference and the evolution of sex. *J. Hered.* **112**, 9–18 (2020).

37. M. J. McDonald, D. P. Rice, M. M. Desai, Sex speeds adaptation by altering the dynamics of molecular evolution. *Nature* **531**, 233–236 (2016).

38. O. Kaltz, G. Bell, The ecology and genetics of fitness in Chlamydomonas. XII. Repeated sexual episodes increase rates of adaptation to novel environments. *Evolution* **56**, 1743–1753 (2002).

39. M. R. Goddard, H. C. J. Godfray, A. Burt, Sex increases the efficacy of natural selection in experimental yeast populations. *Nature* **434**, 636–640 (2005).

40. I. I. Davydov, N. Salamin, M. Robinson-Rechavi, Large-scale comparative analysis of codon models accounting for protein and nucleotide selection. *Mol. Biol. Evol.* **36**, 1316–1332 (2019).

41. W. Haerty, S. Jagadeeshan, R. J. Kulathinal, A. Wong, K. Ravi Ram, L. K. Sirot, L. Levesque, C. G. Artieri, M. F. Wolfner, A. Civetta, R. S. Singh, Evolution in the fast lane: Rapidly evolving sex-related genes in Drosophila. *Genetics* **177**, 1321–1335 (2007).

42. B. Charlesworth, C. H. Langley, The evolution of self-regulated transposition of transposable elements. *Genetics* **112**, 359–383 (1986).

43. T. Schwander, R. Libbrecht, L. Keller, Supergenes and complex phenotypes. *Curr. Biol.* **24**, R288–R294 (2014).

44. D. Bachtrog, Y-chromosome evolution: Emerging insights into processes of Y-chromosome degeneration. *Nat. Rev. Genet.* **14**, 113–124 (2013).

45. R. Riesch, M. Muschick, D. Lindtke, R. Villoutreix, A. A. Comeault, T. E. Farkas, K. Lucek, E. Hellen, V. Soria-Carrasco, S. R. Dennis, C. F. de Carvalho, R. J. Safran, C. P. Sandoval, J. Feder, R. Gries, B. J. Crespi, G. Gries, Z. Gompert, P. Nosil, Transitions between phases of genomic differentiation during stick-insect speciation. *Nat. Ecol. Evol.* **1**, 0082 (2017).

46. S. D. Jackman, B. P. Vandervalk, H. Mohamadi, J. Chu, S. Yeo, S. A. Hammond, G. Jahesh, H. Khan, L. Coombe, R. L. Warren, I. Birol, ABySS 2.0: Resource-efficient assembly of large genomes using a Bloom filter. *Genome Res.* **27**, 768–777 (2017).

47. K. Sahlin, R. Chikhi, L. Arvestad, Assembly scaffolding with PE-contaminated mate-pair libraries. *Bioinformatics* **32**, 1925–1932 (2016).

48. D. R. Laetsch, M. L. Blaxter, BlobTools: Interrogation of genome assemblies. *F1000Res.* **6**, 1287 (2017).

49. D. J. Parker, J. Bast, K. Jalvingh, Z. Dumas, M. Robinson-Rechavi, T. Schwander, Sex-biased gene expression is repeatedly masculinized in asexual females. *Nat. Commun.* **10**, 4638 (2019).

50. D. J. Parker, J. Bast, K. Jalvingh, Z. Dumas, M. Robinson-Rechavi, T. Schwander, Repeated evolution of asexuality involves convergent gene expression changes. *Mol. Biol. Evol.* **36**, 350–364 (2019).

51. B. J. Haas, A. Papanicolaou, M. Yassour, M. Grabherr, P. D. Blood, J. Bowden, M. B. Couger, D. Eccles, B. Li, M. Lieber, M. D. MacManes, M. Ott, J. Orvis, N. Pochet, F. Strozzi, N. Weeks, R. Westerman, T. William, C. N. Dewey, R. Henschel, R. D. LeDuc, N. Friedman, A. Regev, De novo transcript sequence reconstruction from RNA-seq using the Trinity platform for reference generation and analysis. *Nat. Protoc.* **8**, 1494–1512 (2013).

52. C. Holt, M. Yandell, MAKER2: An annotation pipeline and genome-database management tool for second-generation genome projects. *BMC Bioinformatics* **12**, 491 (2011).

53. E. V. Kriventseva, F. Tegenfeldt, T. J. Petty, R. M. Waterhouse, F. A. Simão, I. A. Pozdnyakov, P. Ioannidis, E. M. Zdobnov, OrthoDB v8: Update of the hierarchical catalog of orthologs and the underlying free software. *Nucleic Acids Res.* **43**, D250–D256 (2015).

54. C. M. Francois, F. Durand, E. Figuet, N. Galtier, Prevalence and implications of contamination in public genomic resources: A case study of 43 reference arthropod assemblies. *G3* **10**, 721–730 (2020).

55. B. Buchfink, C. Xie, D. H. Huson, Fast and sensitive protein alignment using DIAMOND. *Nat. Methods* **12**, 59–60 (2015).

56. T. D. Wu, C. K. Watanabe, GMAP: A genomic mapping and alignment program for mRNA and EST sequences. *Bioinformatics* **21**, 1859–1875 (2005).

57. V. Miele, S. Penel, L. Duret, Ultra-fast sequence clustering from similarity networks with SiLiX. *BMC Bioinformatics* **12**, 116 (2011).

58. K. Katoh, D. M. Standley, MAFFT multiple sequence alignment software version 7: Improvements in performance and usability. *Mol. Biol. Evol.* **30**, 772–780 (2013).

59. A. Di Franco, R. Poujol, D. Baurain, H. Philippe, Evaluating the usefulness of alignment filtering methods to reduce the impact of errors on evolutionary inferences. *BMC Evol. Biol.* **19**, 21 (2019).

60. A. Stamatakis, RAxML version 8: A tool for phylogenetic analysis and post-analysis of large phylogenies. *Bioinformatics* **30**, 1312–1313 (2014).

61. G. A. Van der Auwera, M. O. Carneiro, C. Hartl, R. Poplin, G. Del Angel, A. Levy-Moonshine, T. Jordan, K. Shakir, D. Roazen, J. Thibault, E. Banks, K. V. Garimella, D. Altshuler, S. Gabriel, M. A. DePristo, From FastQ data to high confidence variant calls: The Genome Analysis Toolkit best practices pipeline. *Curr. Protoc. Bioinformatics* **43**, 11.10.1–11.10.33 (2013).

62. X. Chen, O. Schulz-Trieglaff, R. Shaw, B. Barnes, F. Schlesinger, M. Källberg, A. J. Cox, S. Kruglyak, C. T. Saunders, Manta: Rapid detection of structural variants and indels for germline and cancer sequencing applications. *Bioinformatics* **32**, 1220–1222 (2016).

63. D. C. Jeffares, C. Jolly, M. Hoti, D. Speed, L. Shaw, C. Rallis, F. Balloux, C. Dessimoz, J. Bähler, F. J. Sedlazeck, Transient structural variations have strong effects on quantitative traits and reproductive isolation in fission yeast. *Nat. Commun.* **8**, 14061 (2017).

64. S. Kurtz, A. Phillippy, A. L. Delcher, M. Smoot, M. Shumway, C. Antonescu, S. L. Salzberg, Versatile and open software for comparing large genomes. *Genome Biol.* **5**, R12 (2004).

65. T. Wicker, F. Sabot, A. Hua-Van, J. L. Bennetzen, P. Capy, B. Chalhoub, A. Flavell, P. Leroy, M. Morgante, O. Panaud, E. Paux, P. SanMiguel, A. H. Schulman, A unified classification system for eukaryotic transposable elements. *Nat. Rev. Genet.* **8**, 973–982 (2007).

66. C. Goubert, L. Modolo, C. Vieira, C. ValienteMoro, P. Mavingui, M. Boulesteix, De novo assembly and annotation of the Asian tiger mosquito (Aedes albopictus) repeatome with dnaPipeTE from raw genomic reads and comparative analysis with the yellow fever mosquito (Aedes aegypti). *Genome Biol. Evol.* **7**, 1192–1205 (2015).

67. R. C. Edgar, Search and clustering orders of magnitude faster than BLAST. *Bioinformatics* **26**, 2460–2461 (2010).

68. C. Hoede, S. Arnoux, M. Moisset, T. Chaumier, O. Inizan, V. Jamilloux, H. Quesneville, PASTEC: An automatic transposable element classification tool. *PLOS ONE* **9**, e91929 (2014).

69. S. F. Altschul, T. L. Madden, A. A. Schäffer, J. Zhang, Z. Zhang, W. Miller, D. J. Lipman, Gapped BLAST and PSI-BLAST: A new generation of protein database search programs. *Nucleic Acids Res.* **25**, 3389–3402 (1997).

70. A. Smit, R. Hubley, P. Green, RepeatMasker Open-4.0 (2013–2015); www.repeatmasker.org.

71. H. Li, Aligning sequence reads, clone sequences and assembly contigs with BWA-MEM. arXiv:1303.3997 [q-bio.GN] (16 March 2013).

72. S. Anders, P. T. Pyl, W. Huber, HTSeq—A Python framework to work with high-throughput sequencing data. *Bioinformatics* **31**, 166–169 (2015).

73. S. Moretti, B. Laurenczy, W. H. Gharib, B. Castella, A. Kuzniar, H. Schabauer, R. A. Studer, M. Valle, N. Salamin, H. Stockinger, M. Robinson-Rechavi, Selectome update: Quality control and computational improvements to a database of positive selection. *Nucleic Acids Res.* **42**, D917–D921 (2014).

74. I. M. Wallace, O. O'Sullivan, D. G. Higgins, C. Notredame, M-Coffee: Combining multiple sequence alignment methods with T-Coffee. *Nucleic Acids Res.* **34**, 1692–1699 (2006).

75. F. Sievers, A. Wilm, D. Dineen, T. J. Gibson, K. Karplus, W. Li, R. Lopez, H. McWilliam, M. Remmert, J. Söding, J. D. Thompson, D. G. Higgins, Fast, scalable generation of high-quality protein multiple sequence alignments using Clustal Omega. *Mol. Syst. Biol.* **7**, 539 (2011).

76. C. Notredame, D. G. Higgins, J. Heringa, T-Coffee: A novel method for fast and accurate multiple sequence alignment. *J. Mol. Biol.* **302**, 205–217 (2000).

77. S. Capella-Gutiérrez, J. M. Silla-Martínez, T. Gabaldón, trimAl: A tool for automated alignment trimming in large-scale phylogenetic analyses. *Bioinformatics* **25**, 1972–1973 (2009).

78. D. Bates, M. Mächler, B. Bolker, S. Walker, Fitting linear mixed-effects models using lme4. *J. Stat. Softw.* **67**, 1–48 (2015).

79. M. Wang, Y. Zhao, B. Zhang, Efficient test and visualization of multi-set intersections. *Sci. Rep.* **5**, 16923 (2015).

80. A. Alexa, J. Rahnenführer, T. Lengauer, Improved scoring of functional groups from gene expression data by decorrelating GO graph structure. *Bioinformatics* **22**, 1600–1607 (2006).

81. A. M. Bolger, M. Lohse, B. Usadel, Trimmomatic: A flexible trimmer for Illumina sequence data. *Bioinformatics* **30**, 2114–2120 (2014).

82. J. O'Connell, O. Schulz-Trieglaff, E. Carlson, M. M. Hims, N. A. Gormley, A. J. Cox, NxTrim: Optimized trimming of Illumina mate pair reads. *Bioinformatics* **31**, 2035–2037 (2015).

83. J. T. Simpson, K. Wong, S. D. Jackman, J. E. Schein, S. J. M. Jones, I. Birol, ABySS: A parallel assembler for short read sequence data. *Genome Res.* **19**, 1117–1123 (2009).

84. R. Chikhi, P. Medvedev, Informed and automated k-mer size selection for genome assembly. *Bioinformatics* **30**, 31–37 (2014).

85. R. Luo, B. Liu, Y. Xie, Z. Li, W. Huang, J. Yuan, G. He, Y. Chen, Q. Pan, Y. Liu, J. Tang, G. Wu, H. Zhang, Y. Shi, Y. Liu, C. Yu, B. Wang, Y. Lu, C. Han, D. W. Cheung, S.-M. Yiu, S. Peng, Z. Xiaoqian, G. Liu, X. Liao, Y. Li, H. Yang, J. Wang, T.-W. Lam, J. Wang, SOAPdenovo2: An empirically improved memory-efficient short-read de novo assembler. *Gigascience* **1**, 18 (2012).

86. E. W. Sayers, J. Beck, J. R. Brister, E. E. Bolton, K. Canese, D. C. Comeau, K. Funk, A. Ketter, S. Kim, A. Kimchi, P. A. Kitts, A. Kuznetsov, S. Lathrop, Z. Lu, K. McGarvey, T. L. Madden, T. D. Murphy, N. O'Leary, L. Phan, V. A. Schneider, F. Thibaud-Nissen, B. W. Trawick, K. D. Pruitt, J. Ostell, Database resources of the National Center for Biotechnology Information. *Nucleic Acids Res.* **48**, D9–D16 (2020).

87. M. Martin, Cutadapt removes adapter sequences from high-throughput sequencing reads. *EMBnet J.* **17**, 10–12 (2011).

88. A. Dobin, C. A. Davis, F. Schlesinger, J. Drenkow, C. Zaleski, S. Jha, P. Batut, M. Chaisson, T. R. Gingeras, STAR: Ultrafast universal RNA-seq aligner. *Bioinformatics* **29**, 15–21 (2013).

89. N. L. Bray, H. Pimentel, P. Melsted, L. Pachter, Near-optimal probabilistic RNA-seq quantification. *Nat. Biotechnol.* **34**, 525–527 (2016).

90. M. S. Campbell, C. Holt, B. Moore, M. Yandell, Genome annotation and curation using MAKER and MAKER-P. *Curr. Protoc. Bioinformatics* **48**, 4.11.1–4.11.39 (2014).

91. M. Stanke, O. Keller, I. Gunduz, A. Hayes, S. Waack, B. Morgenstern, AUGUSTUS: Ab initio prediction of alternative transcripts. *Nucleic Acids Res.* **34**, W435–W439 (2006).

92. UniProt Consortium, UniProt: A worldwide hub of protein knowledge. *Nucleic Acids Res.* **47**, D506–D515 (2019).

93. I. Korf, Gene finding in novel genomes. *BMC Bioinformatics* **5**, 59 (2004).

94. S. Götz, J. M. García-Gómez, J. Terol, T. D. Williams, S. H. Nagaraj, M. J. Nueda, M. Robles, M. Talón, J. Dopazo, A. Conesa, High-throughput functional annotation and data mining with the Blast2GO suite. *Nucleic Acids Res.* **36**, 3420–3435 (2008).

95. A. Conesa, S. Götz, J. M. García-Gómez, J. Terol, M. Talón, M. Robles, Blast2GO: A universal tool for annotation, visualization and analysis in functional genomics research. *Bioinformatics* **21**, 3674–3676 (2005).

96. D. Fontaneto, C. Q. Tang, U. Obertegger, F. Leasi, T. G. Barraclough, Different diversification rates between sexual and asexual organisms. *Evol. Biol.* **39**, 262–270 (2012).

97. E. A. Gladyshev, M. Meselson, I. R. Arkhipova, Massive horizontal gene transfer in bdelloid rotifers. *Science* **320**, 1210–1213 (2008).

98. J.-F. Flot, B. Hespeels, X. Li, B. Noel, I. Arkhipova, E. G. J. Danchin, A. Hejnol, B. Henrissat, R. Koszul, J.-M. Aury, V. Barbe, R.-M. Barthélémy, J. Bast, G. A. Bazykin, O. Chabrol, A. Couloux, M. Da Rocha, C. Da Silva, E. Gladyshev, P. Gouret, O. Hallatschek, B. Hecox-Lea, K. Labadie, B. Lejeune, O. Piskurek, J. Poulain, F. Rodriguez, J. F. Ryan, O. A. Vakhrusheva, E. Wajnberg, B. Wirth, I. Yushenova, M. Kellis, A. S. Kondrashov, D. B. Mark Welch, P. Pontarotti, J. Weissenbach, P. Wincker, O. Jaillon, K. Van Doninck, Genomic evidence for ameiotic evolution in the bdelloid rotifer Adineta vaga. *Nature* **500**, 453–457 (2013).

99. R. W. Nowell, P. Almeida, C. G. Wilson, T. P. Smith, D. Fontaneto, A. Crisp, G. Micklem, A. Tunnacliffe, C. Boschetti, T. G. Barraclough, Comparative genomics of bdelloid rotifers: Insights from desiccating and nondesiccating species. *PLOS Biol.* **16**, e2004830 (2018).

100. E. G. J. Danchin, M.-N. Rosso, P. Vieira, J. de Almeida-Engler, P. M. Coutinho, B. Henrissat, P. Abad, Multiple lateral gene transfers and duplications have promoted plant parasitism ability in nematodes. *Proc. Natl. Acad. Sci. U.S.A.* **107**, 17651–17656 (2010).

101. P. Abad, J. Gouzy, J.-M. Aury, P. Castagnone-Sereno, E. G. J. Danchin, E. Deleury, L. Perfus-Barbeoch, V. Anthouard, F. Artiguenave, V. C. Blok, M.-C. Caillaud, P. M. Coutinho, C. Dasilva, F. De Luca, F. Deau, M. Esquibet, T. Flutre, J. V. Goldstone, N. Hamamouch, T. Hewezi, O. Jaillon, C. Jubin, P. Leonetti, M. Magliano, T. R. Maier, G. V. Markov, P. McVeigh, G. Pesole, J. Poulain, M. Robinson-Rechavi, E. Sallet, B. Ségurens, D. Steinbach, T. Tytgat, E. Ugarte, C. van Ghelder, P. Veronico, T. J. Baum, M. Blaxter, T. Bleve-Zacheo, E. L. Davis, J. J. Ewbank, B. Favery, E. Grenier, B. Henrissat, J. T. Jones, V. Laudet, A. G. Maule, H. Quesneville, M.-N. Rosso, T. Schiex, G. Smant, J. Weissenbach, P. Wincker, Genome sequence of the metazoan plant-parasitic nematode Meloidogyne incognita. *Nat. Biotechnol.* **26**, 909–915 (2008).

102. A. Faddeeva-Vakhrusheva, K. Kraaijeveld, M. F. L. Derks, S. Y. Anvar, V. Agamennone, W. Suring, A. A. Kampfraath, J. Ellers, G. Le Ngoc, C. A. M. van Gestel, J. Mariën, S. Smit, N. M. van Straalen, D. Roelofs, Coping with living in the soil: The genome of the parthenogenetic springtail Folsomia candida. *BMC Genomics* **18**, 493 (2017).

103. G. Schönknecht, A. P. M. Weber, M. J. Lercher, Horizontal gene acquisitions by eukaryotes as drivers of adaptive evolution. *Bioessays* **36**, 9–20 (2014).

104. T. Guo, X.-W. Wang, K. Shan, W. Sun, L.-Y. Guo, The Loricrin-Like Protein (LLP) of phytophthora infestans is required for oospore formation and plant infection. *Front. Plant Sci.* **8**, 142 (2017).

105. Z. Yang, Y. Zhang, E. K. Wafula, L. A. Honaas, P. E. Ralph, S. Jones, C. R. Clarke, S. Liu, C. Su, H. Zhang, N. S. Altman, S. C. Schuster, M. P. Timko, J. I. Yoder, J. H. Westwood, C. W. dePamphilis, Horizontal gene transfer is more frequent with increased heterotrophy and contributes to parasite adaptation. *Proc. Natl. Acad. Sci. U.S.A.* **113**, E7010–E7019 (2016).

106. J. R. Belyeu, M. Chowdhury, J. Brown, B. S. Pedersen, M. J. Cormier, A. R. Quinlan, R. M. Layer, Samplot: A platform for structural variant visual validation and automated filtering. *Genome Biol.* **22**, 161 (2021).

107. M. Mahmoud, N. Gobet, D. I. Cruz-Dávalos, N. Mounier, C. Dessimoz, F. J. Sedlazeck, Structural variant calling: The long and the short of it. *Genome Biol.* **20**, 246 (2019).

108. J. Ruan, H. Li, Fast and accurate long-read assembly with wtdbg2. *Nat. Methods* **17**, 155–158 (2020).

109. F. J. Sedlazeck, P. Rescheneder, M. Smolka, H. Fang, M. Nattestad, A. von Haeseler, M. C. Schatz, Accurate detection of complex structural variations using single-molecule sequencing. *Nat. Methods* **15**, 461–468 (2018).

110. T. S. Korneliussen, A. Albrechtsen, R. Nielsen, ANGSD: Analysis of next generation sequencing data. *BMC Bioinformatics* **15**, 356 (2014).

111. C. Trapnell, A. Roberts, L. Goff, G. Pertea, D. Kim, D. R. Kelley, H. Pimentel, S. L. Salzberg, J. L. Rinn, L. Pachter, Differential gene and transcript expression analysis of RNA-seq experiments with TopHat and Cufflinks. *Nat. Protoc.* **7**, 562–578 (2012).

112. A. Löytynoja, N. Goldman, An algorithm for progressive multiple alignment of sequences with insertions. *Proc. Natl. Acad. Sci. U.S.A.* **102**, 10557–10562 (2005).

113. G. Talavera, J. Castresana, Improvement of phylogenies after removing divergent and ambiguously aligned blocks from protein sequence alignments. *Syst. Biol.* **56**, 564–577 (2007).

114. A. A. Comeault, S. M. Flaxman, R. Riesch, E. Curran, V. Soria-Carrasco, Z. Gompert, T. E. Farkas, M. Muschick, T. L. Parchman, T. Schwander, J. Slate, P. Nosil, Selection on a genetic polymorphism counteracts ecological speciation in a stick insect. *Curr. Biol.* **25**, 1975–1981 (2015).

115. R. Villoutreix, C. F. de Carvalho, V. Soria-Carrasco, D. Lindtke, M. De-la-Mora, M. Muschick, J. L. Feder, T. L. Parchman, Z. Gompert, P. Nosil, Large-scale mutation in the evolution of a gene complex for cryptic coloration. *Science* **369**, 460–466 (2020).

116. M. Petersen, D. Armisén, R. A. Gibbs, L. Hering, A. Khila, G. Mayer, S. Richards, O. Niehuis, B. Misof, Diversity and evolution of the transposable element repertoire in arthropods with particular reference to insects. *BMC Evol. Biol.* **19**, 11 (2019).
